# Screening and validation of platelet activation-related lncRNAs as potential biomarkers for prognosis and immunotherapy in gastric cancer patients

**DOI:** 10.3389/fgene.2022.965033

**Published:** 2022-09-14

**Authors:** Mingjie Yuan, Yanfei Jia, Yuanxin Xing, Yunshan Wang, Yunyun Liu, Xiangdong Liu, Duanrui Liu

**Affiliations:** ^1^ Department of Clinical Laboratory, Shandong Provincial Hospital Affiliated to Shandong First Medical University, Jinan, China; ^2^ Department of Laboratory, Jinan Central Hospital Affiliated to Shandong First Medical University, Jinan, China; ^3^ Research Center of Basic Medicine, Jinan Central Hospital, Shandong First Medical University, Jinan, China; ^4^ Research Center of Basic Medicine, Jinan Central Hospital, Cheeloo College of Medicine, Shandong University, Jinan, China

**Keywords:** gastric cancer, immunotherapy, platelet, lncRNA, prognosis

## Abstract

**Background:** Platelets (PLT) have a significant effect in promoting cancer progression and hematogenous metastasis. However, the effect of platelet activation-related lncRNAs (PLT-related lncRNAs) in gastric cancer (GC) is still poorly understood. In this study, we screened and validated PLT-related lncRNAs as potential biomarkers for prognosis and immunotherapy in GC patients.

**Methods:** We obtained relevant datasets from the Cancer Genome Atlas (TCGA) and Gene Ontology (GO) Resource Database. Pearson correlation analysis was used to identify PLT-related lncRNAs. By using the univariate, least absolute shrinkage and selection operator (LASSO) Cox regression analyses, we constructed the PLT-related lncRNAs model. Kaplan-Meier survival analysis, univariate, multivariate Cox regression analysis, and nomogram were used to verify the model. The Gene Set Enrichment Analysis (GSEA), drug screening, tumor immune microenvironment analysis, epithelial-mesenchymal transition (EMT), and DNA methylation regulators correlation analysis were performed in the high- and low-risk groups. Patients were regrouped based on the risk model, and candidate compounds and immunotherapeutic responses aimed at GC subgroups were also identified. The expression of seven PLT-related lncRNAs was validated in clinical medical samples using quantitative reverse transcription-polymerase chain reaction (qRT-PCR).

**Results:** In this study, a risk prediction model was established using seven PLT-related lncRNAs -(AL355574.1, LINC01697, AC002401.4, AC129507.1, AL513123.1, LINC01094, and AL356417.2), whose expression were validated in GC patients. Kaplan-Meier survival analysis, the receiver operating characteristic (ROC) curve analysis, univariate, multivariate Cox regression analysis verified the accuracy of the model. We screened multiple targeted drugs for the high-risk patients. Patients in the high-risk group had a poorer prognosis since low infiltration of immune killer cells, activation of immunosuppressive pathways, and poor response to immunotherapy. In addition, we revealed a close relationship between risk scores and EMT and DNA methylation regulators. The nomogram based on risk score suggested a good ability to predict prognosis and high clinical benefits.

**Conclusion:** Our findings provide new insights into how PLT-related lncRNAs biomarkers affect prognosis and immunotherapy. Also, these lncRNAs may become potential biomarkers and therapeutic targets for GC patients.

## Introduction

According to the most recent statistics from the American Cancer Society, the quantity of new cases and deaths cases of gastric cancer (GC) still remain a high level, and GC is the most common malignant tumor of the digestive system (Cao et al., 2020; Siegel et al., 2021). Although the 5-year survival rate of patients with early GC can reach more than 90%, due to the lack of effective biomarkers and specific clinical appearances, GC patients often present with an advanced-stage tumor at the time of diagnosis (Song et al., 2017), losing their chance to undergo surgery (Tan, 2019). Therefore, the search for new cancer-related prognostic molecular biomarkers and new targets is still needed to enhance the individual evaluation and survival rate of GC.

Studies have indicated that platelet (PLT) regulates tumorigenesis and tumor progression, such as GC, prostate cancer, lung cancer, breast cancer, and colorectal cancer *etc* (Oh et al., 2019; Garmi et al., 2020; Meikle et al., 2020; Plantureux et al., 2020; Rudzinski et al., 2020; Singla et al., 2020; Wang et al., 2020). For examples, PLT directly promote epithelial-mesenchymal transformation (EMT) of malignant tumors by producing TGF-β, leading to poor prognosis (Labelle et al., 2011; Heldin et al., 2012; Guo et al., 2019; Chong et al., 2020). In tumor angiogenesis, PLT can also produce vascular endothelial growth factor (VEGFR) and promote angiogenesis, providing oxygen and nutrients to malignant cells (Sabrkhany et al., 2011). PLT-produced particles contain a large number of bioactive substances that promote tumor progression. On the contrary, tumor cells can also produce a variety of bioactive substances, such as ADP, TL-6, and TGF-α, to promote PLT activation, and the activated PLT gather around tumor cells and promote tumor progression and metastasis, resulting in a vicious cycle (Schlesinger, 2018). In addition, Zaslavsky *et al.* found that PLT-generated PD-L1 can induce tumor cells that do not express PD-L1 to avoid being cleared by T cells and evade immune surveillance, thus leading to the progression of malignant tumors (Zaslavsky et al., 2020). All this evidence indicates that PLT have prognostic and immunotherapeutic values.

Long non-coding RNAs (lncRNAs) are about 200 nt or more non-coding protein RNAs, which significantly affect tumor immunity (Chandra Gupta and Nandan Tripathi, 2017). Recently, Ye *et al.* suggested using circulating lncRNAs between tumor-educated platelets (TEPs) and serum can be used as a potential diagnostic and discriminative biomarkers for colorectal cancer (Ye et al., 2022). Bioinformatics research indicated that the dysregulation of PLT-related genes is involved in cancer (Xie et al., 2021). Yet, the specific effect of platelet activation-related lncRNAs (PLT-related lncRNAs) is still unclear. Exploring the effect and mechanism of PLT-related lncRNAs in the development and progression of GC may help predict prognosis and therapy targets.

**FIGURE 1:**
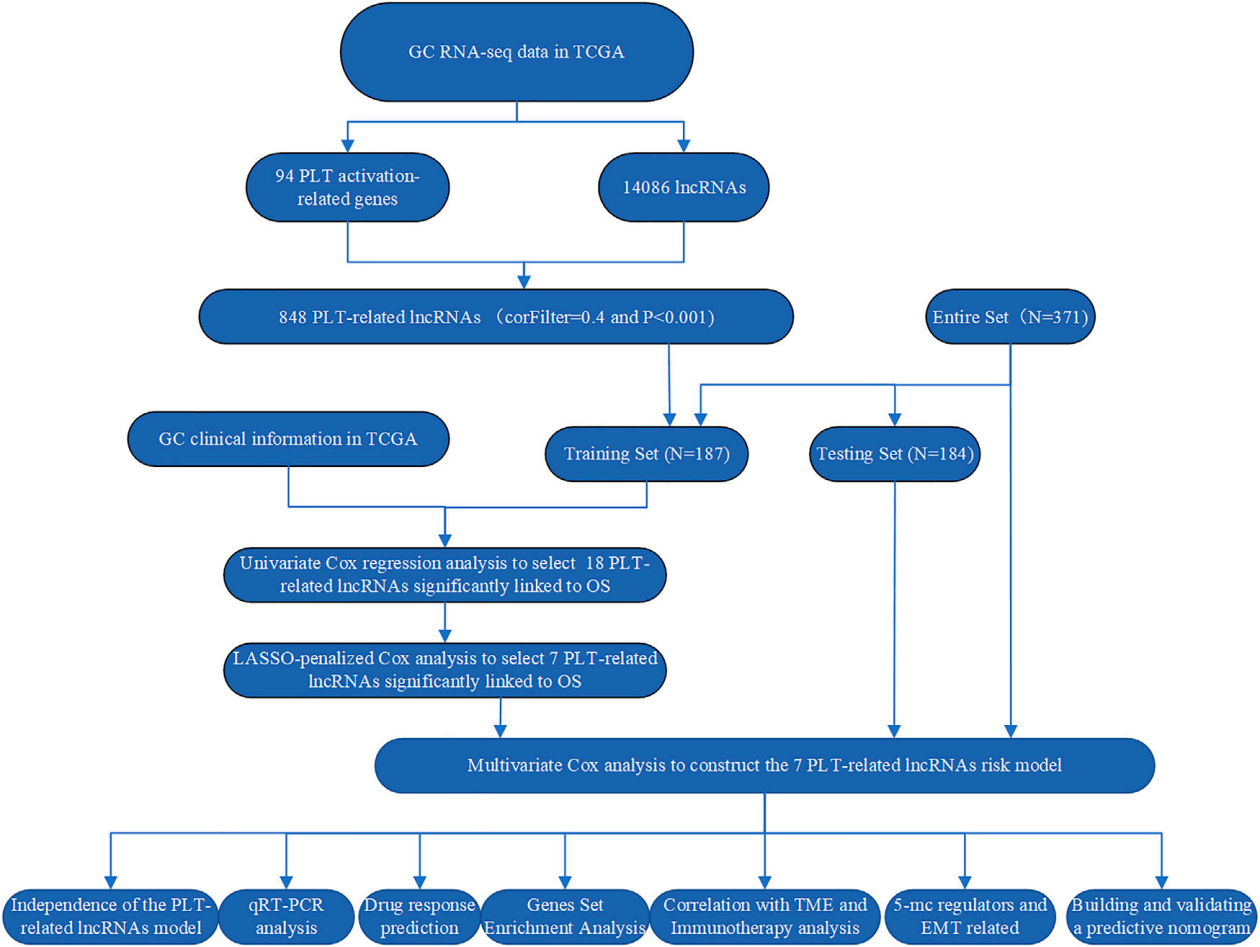
The main process of this study.

In this study, we first extracted 14,087 lncRNAs expression matrix of GC patients from the Cancer Genome Atlas (TCGA) database, and ninety-four genes related to PLT activation were extracted from the Gene Ontology (GO) Resource Database. Then, bioinformatics analysis was performed to identify PLT-related lncRNAs using Pearson’s correlation analysis, after which prognostic risk models were established, and related signaling pathways were screened. Then, we screened for candidate drugs through the Connectivity Map (CMap) database. In addition, we explored the relationship between EMT markers, DNA methylation regulators, and immunotherapy responses and the risk model. Finally, we constructed a nomogram that can predict the overall survival (OS) of GC patients. The study workflow showed in Figure 1.

## Materials and methods

### Data and samples collection

A total of 417 cases (375 cases of gastric cancer group and 32 cases of normal tissue) with clinical data and RNA sequencing dataset were downloaded from the Cancer Genome Atlas (TCGA). lncRNAs and mRNAs were classified by the Ensemble Human Genome browser GRCh38.p13. Ten gastric cancer and adjacent tissue specimens (specimen collection time: June 2021 to December 2021) were additionally collected from Jinan Central Hospital affiliated to Shandong First Medical University, which had been approved by the Ethics Committee of Jinan Central Hospital affiliated to Shandong First Medical University, and all patients had signed informed consent. Supplementary Table S1 shows the clinicopathological characteristics of the included patients. Upon collection, fresh tumor and adjacent normal tissues were snap frozen and stored at −80°C until they were taken out. The data from TCGA is public and therefore does not require ethical approval from the relevant authorities.

### Identification of PLT activation-related lncRNAs

A total of 94 genes related to PLT activation were collected from the Gene Ontology (GO) Resource database (http://geneontology.org/). For the purpose of evaluating the relationship between PLT activation genes and lncRNAs, Pearson correlation analysis was conducted with R software (R 4.2.1), and the intersection of lncRNA expression in GC patients with a correlation coefficient of 0.4 and *p* value <0.001 was obtained. A total of 848 lncRNAs associated with PLT activation and their co-expression networks were obtained using “limma” package (Supplementary Figure S1) (Ritchie et al., 2015).

### Construction and validation of PLT-related risk model

We combined lncRNAs expression with survival data using “limma” packages to obtain a prognostic lncRNAs expression matrix associated with PLT activation (*p* < 0.05). Using “survival” package and pfilter = 0.05, univariate Cox analysis showed that 18 PLT-related lncRNAs were significantly correlated with OS (Simon et al., 2011). The “ggpubr” package was then used for differential analysis to obtain the related heatmaps of lncRNAs expression levels in normal and tumor tissues (Whitehead et al., 2019). Lasso regression was performed on these prognostic lncRNAs, and seven lncRNAs associated with PLT in GC were extracted to construct a prognostic risk model (Simon et al., 2011). After excluding normal patients and patients with incomplete clinical data, 372 patients with GC were randomly divided into a testing group and a training group. We used the following algorithm to calculate the risk score for each patient:

Risk Score=∑coef(lncRNA)×expression(lncRNA)

where coef (lncRNA) represents the prognostic lncRNAs coefficient, and expression (lncRNA) indicates the expression level of lncRNAs (Huang et al., 2021a). GC patients were divided into high- and low-risk groups based on the median risk score. Kaplan-Meier survival analysis used “survival” and “survminer” R package (The R Foundation for Statistical Computing, Vienna, Austria) to estimate the survival difference between the two groups. Then we used the receiver operating characteristic (ROC) curves to evaluate the accuracy of the model (Kim and Hwang, 2020).

### Drug screening in risk model

Based on risk scores, effective medicine was screened using CMap (http://portals.broadinstitute.org/camp/) to obtain drugs that reduce risk in high-risk patients (Subramanian et al., 2017). Enrichment score >0 indicated that drugs couldn promote the expression of high-risk genes; a score <0, showed that drugs could suppress the expression of high-risk genes, and *p* < 0.05 showed that drugs could be significantly enriched (Gns et al., 2019). PubChem website (https://pubchem.ncbi.nim.nih.gov/) was used to obtain the molecular structure of the effective drugs (Kim et al., 2021).

### Gene set enrichment analysis

To reveal Gene expression data by sequencing the degree of difference between genes in two groups of samples by using the Gene Set Enrichment Analysis (GSEA) (Subramanian et al., 2005). GC patients were divided into high- and low-risk groups based on the median risk score. For studying the differences in biological functions between risk groups, the Kyoto Encyclopedia of Genes and Genomes (KEGG) pathway enrichment analysis was conducted with GSEA software, and the pathways enriched by high- and low-risk genes were obtained, respectively. *FDR* < 0.25 or *p* < 0.05 were considered statistically significant.

### Estimation of the tumor microenvironment using the PLT-related lncRNAs model

Since GSEA results are mostly immune-related, we planned to analyze the tumor microenvironment (TME) in risk model we constructed. CIBERSORT was used to count the immune infiltration statuses of GC patients (Chen et al., 2018). Differences in the content of 22 types of immune cells of high- and low-risk groups were analyzed by the “vioplot” R package (Hu, 2020). Then “ggpubr” package was used to analyze the differences in the TME scores (Estimate-Scores, Immunity-Scores, and Stromal-Scores) of patients in different risk groups, and patients with high TME scores have poorer prognosis (Yoshihara et al., 2013). Exploring the immunotherapy for the model’s applicability can promoting more effective immunotherapy strategies. Then, we analyzed the microsatellite instability (MSI) status (MSS, MSI-H, and MSI-L) of the high- and low-risk groups. MSI status files are from TCIA (http://tcia.at). Also, TIDE (http://tide.dfc-i.harvard.edu/) algorithm was used to assess the different responses to immune checkpoint inhibitors in high- and low-risk groups. When the TIDE score increased, tumors were more likely to have immune escape (Jiang et al., 2018).

### Acquisition of DNA methylated regulators and EMT markers

A 5-methylcytosine (5mC) methylated regulator was used to assess the correlation between risk models and DNA methylation. Eleven methylated tuning nodes were obtained from the literature (Chen et al., 2020). EMT-associated genes were used to evaluate the relationship between EMT and the risk model. EMT-related genes were obtained from the EMTome website (Vasaikar et al., 2021). We selected the top 10 markers from the EMTome website for which we could find the expression level for correlation analysis.

### Quantitative reverse transcription-polymerase chain reaction analysis

Total RNA was extracted from 10 gastric cancer patients by TRIzon method. cDNA synthesis was then performed using reverse transcription reagents. Quantitative reverse transcription-polymerase chain reaction (qRT-PCR) was performed using 2× SYBR Green HS Premix (AG) on Roche 480 instrument with β-actin as an internal reference. Gene expression levels were calculated using the 2-ΔΔCT method (Livak and Schmittgen, 2001). Supplementary Table S2 shows the primer sequences used to amplify the seven lncRNAs.

### Statistical analysis

R software 4.1.2 and GraphPad Prism 8 were used to analyze the data. The R software package “survival” and “survminer” were used for univariate Cox proportional risk regression analysis, multivariate Cox proportional risk regression analysis, and nomogram analysis. The Wilcoxon rank sum test or Kruskal–Wallis rank sum test was used to analyze the differences between the two groups; logarithmic rank testing was used to calculate the statistical difference of OS between high-and low-risk group. The R software package “glmnet” was used for lasso Cox proportional regression, and the R package “survival ROC” was used as the ROC curve (Simon et al., 2011). The *p* value <0.05 was considered to be statistically significant.

## Results

### Identification of PLT-related lncRNA of GC patients

**FIGURE 2:**
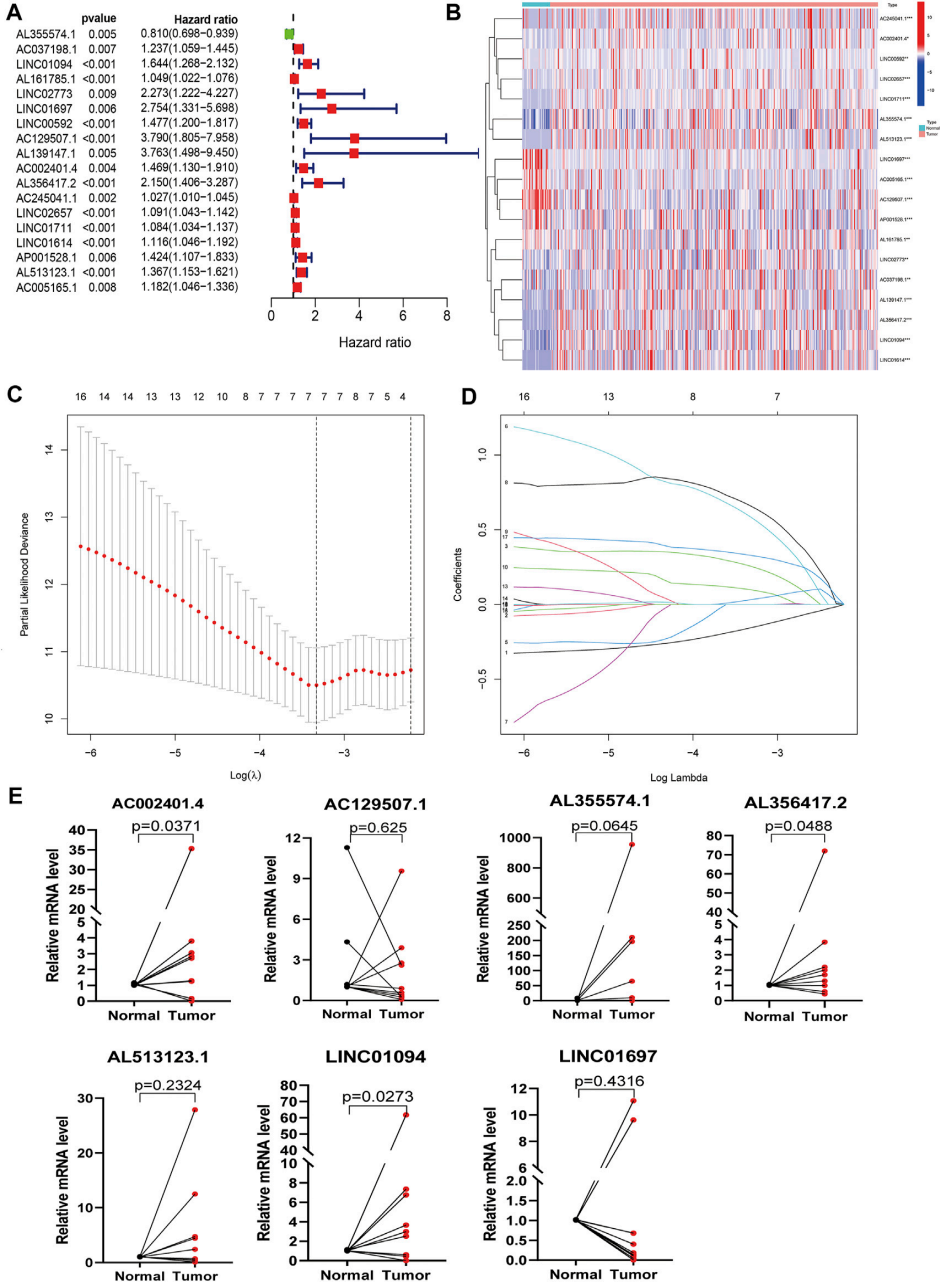
Identification of PLT-related lncRNAs in patients with GC. **(A)** Univariate Cox regression analysis was used to extract the prognostic lncRNAs. **(B)** Heatmaps of 18 prognostic lncRNAs expression of patients (***p < 0.001 * *p < 0.01 *p < 0.05). **(C)** The LASSO coefficient profile of 18 PLT-related lncRNAs. **(D)** The LASSO coefficient distributions of OS-related lncRNAs and vertical dashed lines were plotted with the values selected for 10x cross-validation. **(E)** The results of qRT-PCR of PLT-related lncRNAs of 10 pairs GC patients.

Figure 1 shows the detailed workflow of the study. Firstly, 94 PLT activation-related genes were extracted from the Gene Ontology (GO) Resource Database (Supplementary Table S3), and 14,086 lncRNAs expression matrices were extracted from GC from the TCGA database. PLT-related lncRNAs were defined as those that were significantly correlated with one of the 94 PLT-related genes (|PearsonR| > 0.4 and *p* < 0.001). PLT-related genes and lncRNAs co-expression network is shown in Supplementary Figure S1, and 848 PLT-related lncRNAs were identified (Supplementary Table S3). Eighteen PLT-related lncRNAs were significantly correlated with OS by using univariate Cox regression analysis (Figure 2A). Then, we analyzed the expression levels of these lncRNAs in GC and corresponding normal tissues (Figure 2B). The results showed that among the 18 PLT-related lncRNAs, most lncRNAs (AL355574.1, AC037198.1, LINC01094, LINC02773, LINC00592, AL139147.1, AC002401.4, AL356417.2, AC245041.1, LINC02657, AL139147.1, AC002401.4, AL356417.2, LINC02657, LINC02657, AL355574.1, AC037198.1, LINC01094, LINC02773, LINC00592, AL139147.1, AC002401.4, LINC01711, LINC01614, AL513123.1) were up-regulated and four lncRNAs (AL161785.1, LINC01697, AC129507.1, AP001528.1, AC005165.1) were down-regulated in GC compared to the normal tissues (Figure 2B, *p* < 0.05). LASSO-penalized Cox analysis was then performed on the 18 lncRNAs, and vertical dashed lines were drawn at the optimal value when the order of Log(λ) was the least likely deviation for OS-related adjustment parameters, and seven lncRNAs related to the prognosis of PLT activation in GC were extracted (Figures 2C,D). These seven PLT-related lncRNAs (AL355574.1, LINC01697, AC002401.4, AC129507.1, AL513123.1, LINC01094, AL356417.2) were used to build a risk model to evaluate the prognostic risk of GC patients.

In addition, we further verified model-related seven lncRNAs (AL355574.1, LINC01697, AC002401.4, AC129507.1, AL513123.1, LINC01094, AL356417.2) in GC patient tissues and corresponding adjacent tissues using qRT-PCR. We observed that the expression levels of AL355574.1, AC002401.4, LINC01094, and AL356417.2 were up-regulated in most GC tissues, while LINC01697 and AC129507.1 were down-regulated (Figure 2E), which is consistent to the results of TCGA data.

### Construction and validation of risk model based on PLT-related lncRNAs

**FIGURE 3:**
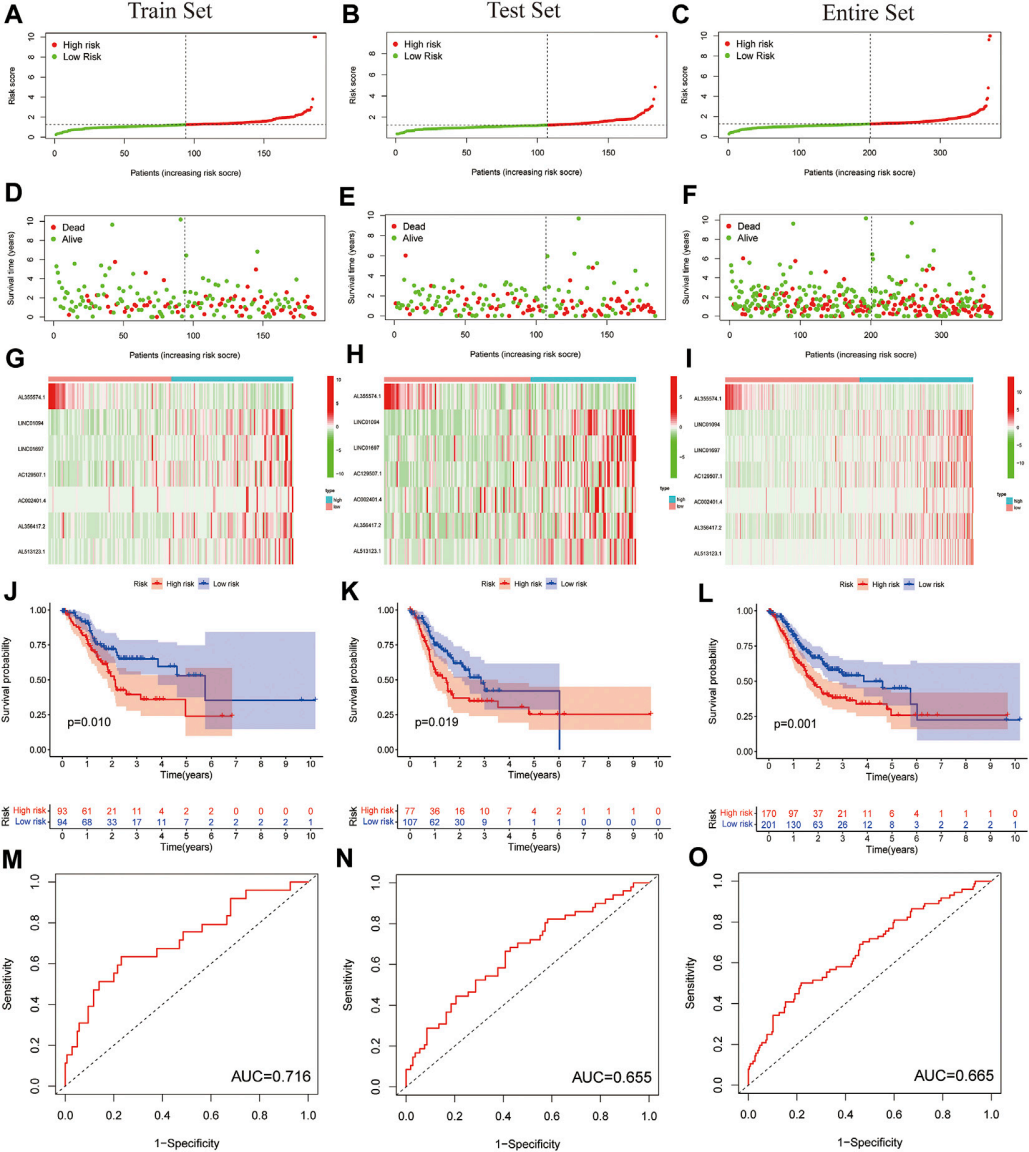
Prognostic value of the risk model of the seven PLT-related lncRNAs in the testing set and training set. **(A**–**C)** Distribution of PLT-related lncRNAs model presented based on a training set, testing set, and entire set risk scores. **(D**–**F)** Survival time and survival status of low- and high-risk groups for the training set, testing set and entire set. **(G**–**I)** Heat-maps of seven LncRNA expressions in the training set, testing set, and entire set. **(J**–**L)** Kaplan-Meier survival curves of the OS of patients in the training set, testing set, and entire set. **(M**–**O)** ROC curve of the training set, testing set, and entire set.

For further testing, the predictive value of the model and the risk scores for each patient were calculated by using a unified formula. Patients were divided into testing set and training set for analysis and validation. Then, based on the median risk score, patients were divided into high- and low-risk groups. The distribution of clinical characteristics of patients in each group is shown in Table 1. The distribution of PLT-related lncRNAs risk scores in the training set and testing set are shown in Figures 3A,B. There were significant differences in the living conditions in survival status among different risk groups. Red dots indicate death and green dots indicate survival. Many cases died in the high-risk group, while most patients in the low-risk group survived (Figures 3D,E). Heatmaps showed seven prognostic lncRNAs expressions for each patient (Figures 3G,H). Figure 3C depicts the distribution of risk levels across all samples for the entire set. The survival status and duration of patients in the entire set are shown in Figure 3F. The prognostic value expression criteria for seven PLT-related lncRNAs risk patterns per patient in the risk model are shown in Figure 3I. Survival analysis of the training set and testing set showed that the high-risk group had a significantly lower survival rate than the low-risk group (Figures 3J,K, *p* < 0.05). However, the survival analysis of the entire set showed the same results (Figure 3L). ROC curve analysis was used to assess the accuracy of the prognostic model. The results showed that the area under the ROC curve (AUC) of the training set was 0.716 (Figure 3M), the AUC of the testing set was 0.655 (Figure 3N), and the entire set was 0.665 (Figure 3O). The ROC analysis results suggested that the risk model we constructed has high reliability (AUC>0.5). Collectively, these results suggested the good performance of the risk model for survival prediction.

**TABLE 1:** Distribution of patients’ characteristics.

	Entire set		Train set		Test set	
Characteristics	Number	Percentage	Number	Percentage	Number	Percentage
Age						
<60 years	111	29.92	57	30.48	54	29.35
≥60 years	257	69.27	128	68.45	129	70.11
Not available	3	0.81	2	1.07	1	0.54
Gender						
Female	133	35.85	66	35.29	67	36.41
Male	238	64.15	121	64.71	117	63.59
Grade						
Grade 1–2	144	38.81	71	37.96	73	39.67
Grade 3	218	58.76	115	61.5	103	55.98
Not available	9	2.43	1	0.54	8	4.35
Stage						
Stage I–II	161	43.4	87	46.52	74	40.22
Stage III–IV	187	50.4	91	48.66	96	52.17
Not available	23	6.2	9	4.82	14	7.61
T						
T1–T2	96	25.88	53	28.34	43	23.37
T3–T4	267	72	132	70.59	135	73.37
Not available	8	2.12	2	1.07	6	3.26
M						
M0	328	88.41	166	88.77	162	88.04
M1	25	6.74	10	5.35	15	8.15
Not available	18	4.85	11	5.88	7	3.81
N						
N0	108	29.11	58	31.02	50	27.17
N1–3	245	66.03	120	64.17	125	67.94
Not available	18	4.86	9	4.81	9	4.89

**FIGURE 4:**
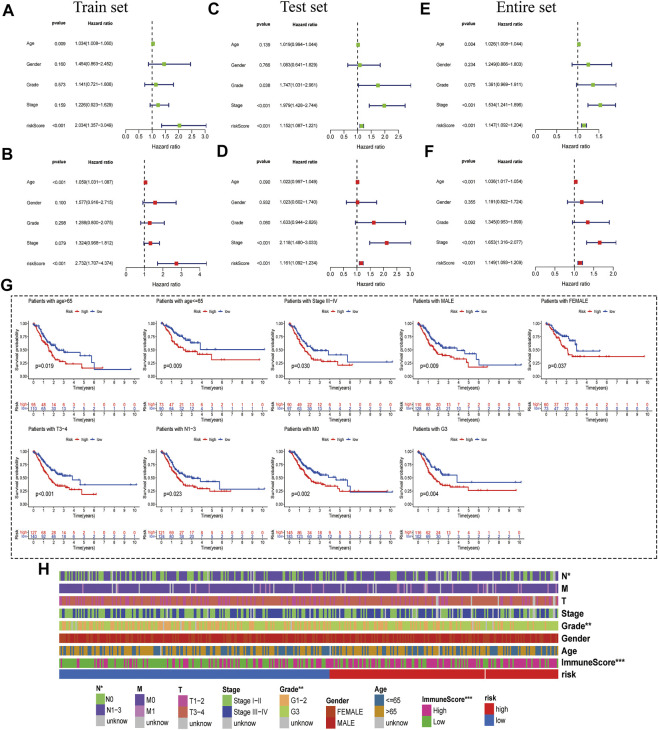
Correlation Analysis between risk score and Clinicopathological Features. **(A**,**B)** Univariate- and multivariate-Cox analyses of clinical characteristics and risk score with OS of the training set. **(C**,**D)** Univariate- and multivariate-Cox analyses of clinical characteristics and risk score with OS of the testing set. **(E**,**F)** Univariate- and multivariate-Cox analyses of clinical characteristics and risk score with OS of the entire set. **(G)** Kaplan-Meier survival curves of the OS of patients between the risk model and clinical characteristics (age, sex, TNM stage, grade, and survival status). **(H)** Heat-map of correlation between high- and low-risk and patient clinical characteristics (***p < 0.001 * *p < 0.01 *p < 0.05).

Then, we conducted univariate and multivariate Cox regression analyses to study whether the prognostic characteristics were independent risk factors. The univariate Cox regression hazard ratio (HR) and 95% confidence interval (CI) of the training set were 2.034 and 1.357–3.049 (*p* < 0.001); in the testing set, HR was 1.152,95% CI was 1.087–1.221 (Figures 4A,C). HR and 95% CI of multivariate Cox regression in the training set were 2.734 and 1.707–4.374 (*p* < 0.001) respectively; HR was 1.161, 95% CI was 1.092–1.234 (*p* < 0.001) in the testing set (Figures 4B,D). For the entire set, we acquired similar results (Figures 4E,F). This result indicated that the risk model was an independent prognostic factor that was not correlated with clinicopathological parameters such as gender, age, tumor grade, and tumor stage.

### Correlation analysis between risk score and clinicopathological features

Based on the TCGA clinical data, differences in OS stratified by common clinicopathological features were analyzed between the low-risk and high-risk groups. In subgroups divided by gender, age, stage, or tumor stage, the OS of the low-risk group was significantly better than that of the high-risk group (Figure 4G and Supplementary Figure S2A). In addition, OS difference curves were stratified between high-risk and low-risk groups by age, gender, tumor grade, or TNM stage. Risk and clinical correlation heatmap showed that risk score is related to Grade, N (*p* = 0.0087) and immune score (*p* < 0.001), but not to age, gender, and TM stage, Stages (*p* < 0.05) (Figure 4H and Supplementary Figure S2B).

### Identification of drugs targeting PLT-related lncRNAs model

In order to determine the effective drug for the PLT-related lncRNAs model, we used the CMap drug screening website (https://portals.broadinstitute.org/cmap/). For enrichment scores, negative values indicate that the drugs can inhibit the expression of high-risk genes and improve the survival rate of patients. Positive values represent that it can promote the expression of high-risk genes (Gns et al., 2019). Seventy compounds were screened out (*p* < 0.05). All screened compounds could reduce the death risk in high-risk patients, and thus deserve further analysis in GC patients (Table 2). The secondary structure and tertiary structure of some drugs are shown in Supplementary Figure S3.

**TABLE 2:** The compounds screened that can reduce GC patients’ risk.

Rank	Cmap name	Mean	*n*	Enrichment	*p*
1	heptaminol	−0.331	5	−0.842	0.00026
2	etiocholanolone	−0.36	6	−0.738	0.00068
3	trimethobenzamide	−0.37	5	−0.791	0.00078
4	thapsigargin	−0.61	3	−0.893	0.00236
5	sulfamonomethoxine	−0.273	4	−0.8	0.0031
6	pheneticillin	−0.296	4	−0.777	0.00511
7	amantadine	−0.263	4	−0.767	0.00599
8	colistin	−0.448	4	−0.764	0.00635
9	3-acetamidocoumarin	−0.35	4	−0.762	0.00656
10	alprostadil	−0.231	7	−0.578	0.00976
11	N-acetylmuramic acid	−0.533	4	−0.715	0.01333
12	Prestwick-1103	−0.45	4	−0.701	0.01671
13	pyrithyldione	−0.462	4	−0.677	0.02397
14	vincamine	−0.294	6	−0.564	0.02543
15	Prestwick-857	−0.281	4	−0.661	0.02988
16	terazosin	−0.332	4	−0.655	0.03284
17	aconitine	−0.281	4	−0.648	0.03638
18	indoprofen	−0.431	4	−0.639	0.04048
19	acebutolol	−0.385	5	−0.577	0.04075
20	2-aminobenzenesulfonamide	−0.326	4	−0.631	0.04538
21	nifuroxazide	−0.247	4	−0.63	0.04611
22	nimodipine	−0.187	4	−0.625	0.0487

### Pathway enrichment analysis

**FIGURE 5:**
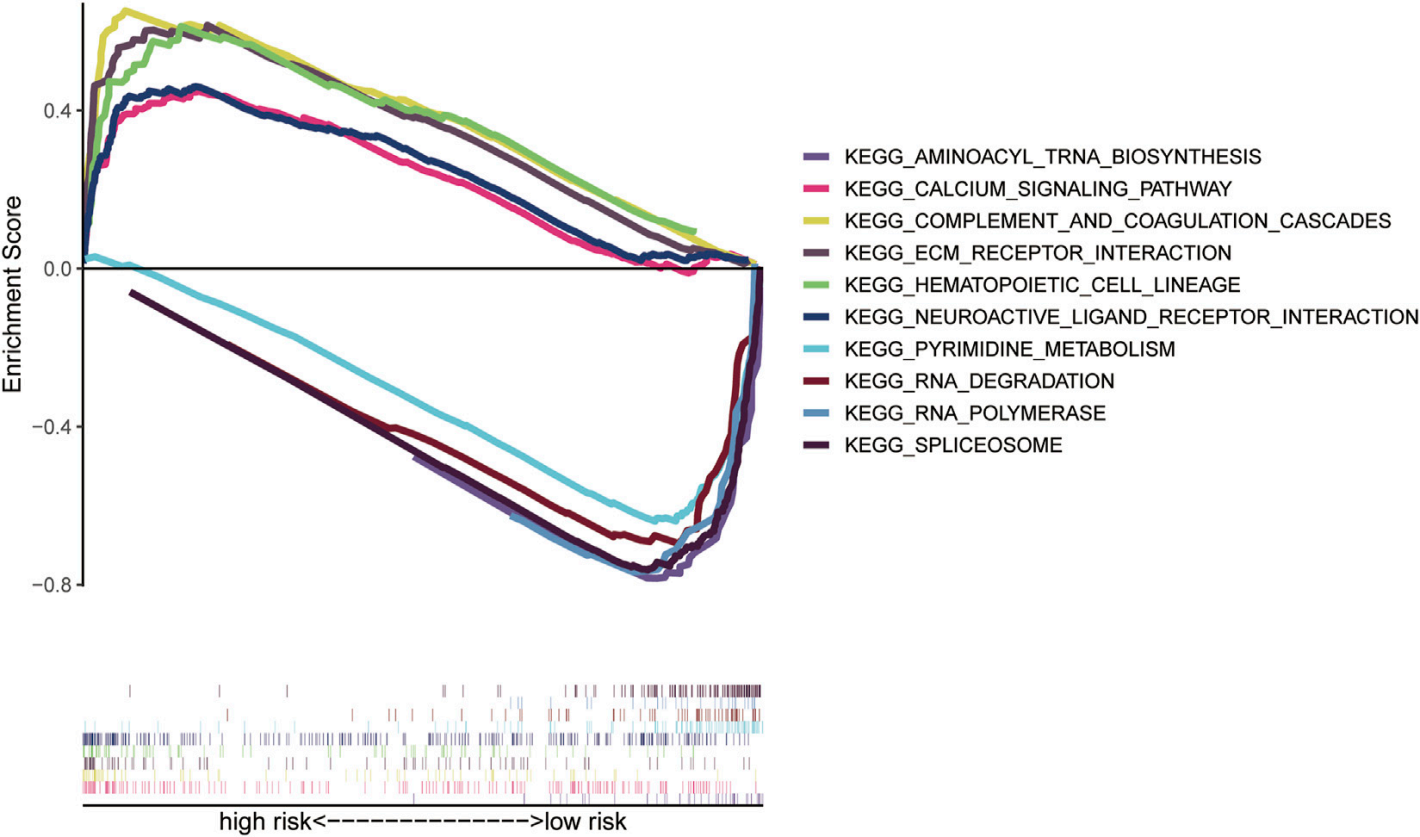
Pathway Enrichment Analysis. **(A)** GSEA analysis of the high- and low-risk groups.

To further explore the potential molecular mechanism of PLT-related lncRNAs and study the differences in biological functions between risk groups, each clinical sample was divided into high-risk (C2) and low-risk (C1) groups. Then, KEGG pathway enrichment analysis was performed with GSEA software. The pathways enriched by high- and low-risk genes were obtained, respectively. Pathways enriched in the C2 group mainly included complement and coagulation cascades, hematopoietic cell lineage, neuroactive ligand-receptor interaction, ECM receptor interaction, and other signaling pathways (Supplementary Figure S4). The pathways enriched in the C1 group mainly included spliceosome, RNA degradation, RNA polymerase, spliceosome, neuroactive tRNA biosynthesis, base excised repair, nucleotide excised repair, homologous recombination, P53 signaling pathway, *et al.* (Supplementary Figure S4). Figure 5 shows the top five pathways with the highest correlation in the high- and low-risk group. Details of the GSEA results are listed in Supplementary Tables S4, S5. We found that the high-risk group had more pathways related to immunosuppression, such as extracellular matrix (ECM) receptor interaction, which is a complex network of ECM molecules (Zeltz et al., 2020).

### TME and immunotherapy response evaluation using risk model

**FIGURE 6:**
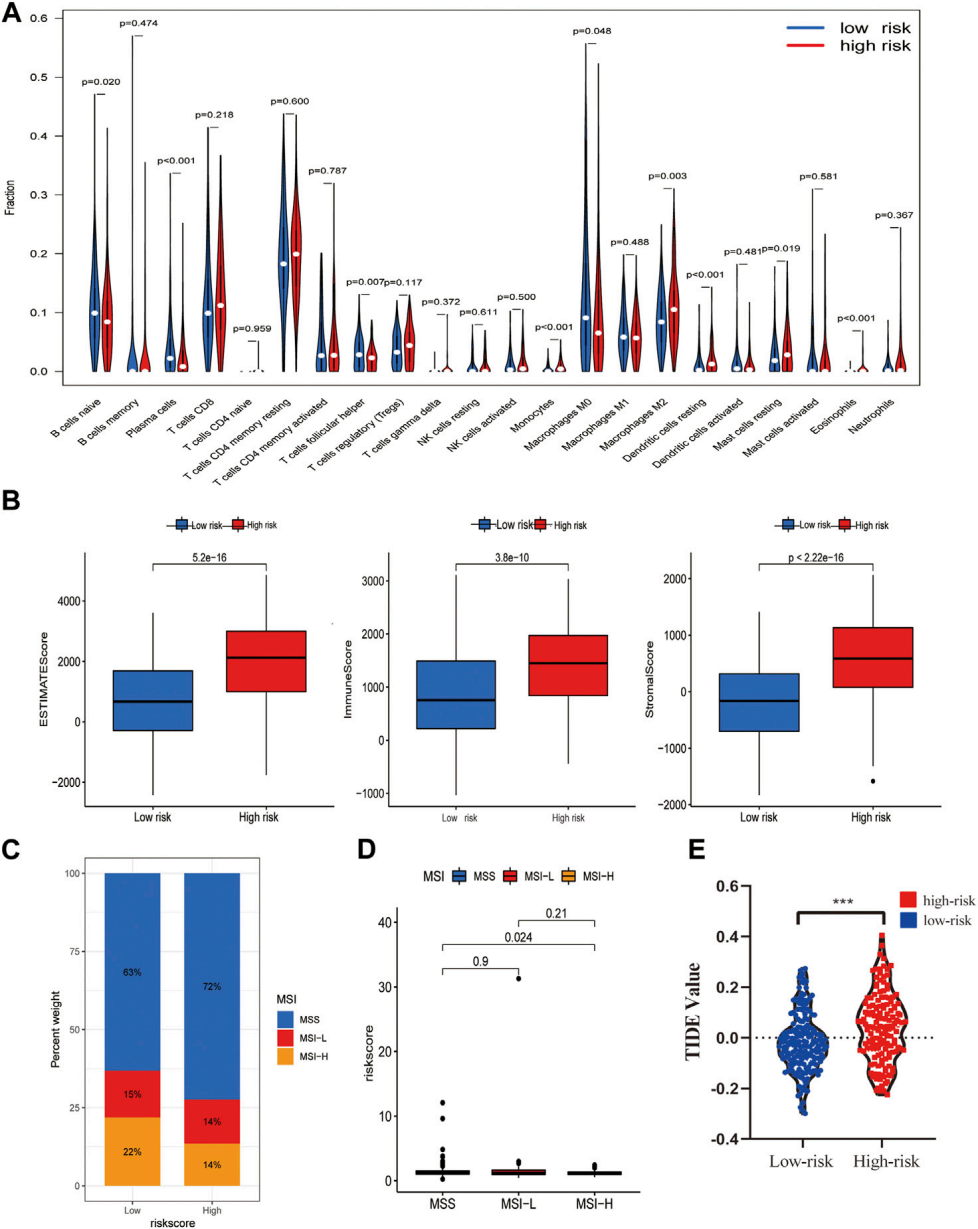
Potential role of the risk model in the TME and immunotherapy. **(A)** The content of 22 immune cells between the high- and low-risk groups. **(B)** TME Estimate-Scores, Immunity-Scores, and Stromal-Scores measured between high- and low-risk groups. **(C**,**D)** Differences in microsatellite instability (MSI) between patients in the high- and low-risk groups. **(E)** Differences in immunotherapy scores between high- and low-risk groups.

CIBERSOPT was used to analyze the correlation between TME and tumor immunotherapy in the PLT-related lncRNA model. Next, we analyzed the differences of 22 immune cell subtypes in the high- and low-risk groups (Figure 6A). Lower-risk patients had higher enrichment levels of immune killer cells. For example, B cells naive, Plasma cells, T cells follicular helper T cells regulatory, and Macrophages M0 cells were significantly increased in the low-risk group (Figure 6A). In addition, we also validated the correlation of the risk model with immune cells using other algorithms (Supplementary Figure S5). The results of the TME scores assessment showed that indicated that the immune, stromal, and estimate scores of the high-risk group were higher than the low-risk group (*p* < 0.05) (Figure 6B). Besides, more and more studies show that microsatellite instability (MSI) status affects the TME and patients with microsatellite instability-high (MSI-H) are more sensitive to immunotherapy (Lin et al., 2020). Our studies showed that low-risk scores were associated with MSI-H, which predicted that low-risk patients are more likely to benefit from immunotherapy (Figures 6C,D). Furthermore, differences in TIDE scores between high- and low-risk groups were obtained, and the results showed high-risk patients had higher TIDE scores, predicting poorer immunotherapy outcomes (Figure 6E).

### Correlation analysis of PLT-related lncRNAs with DNA methylation and EMT

**FIGURE 7:**
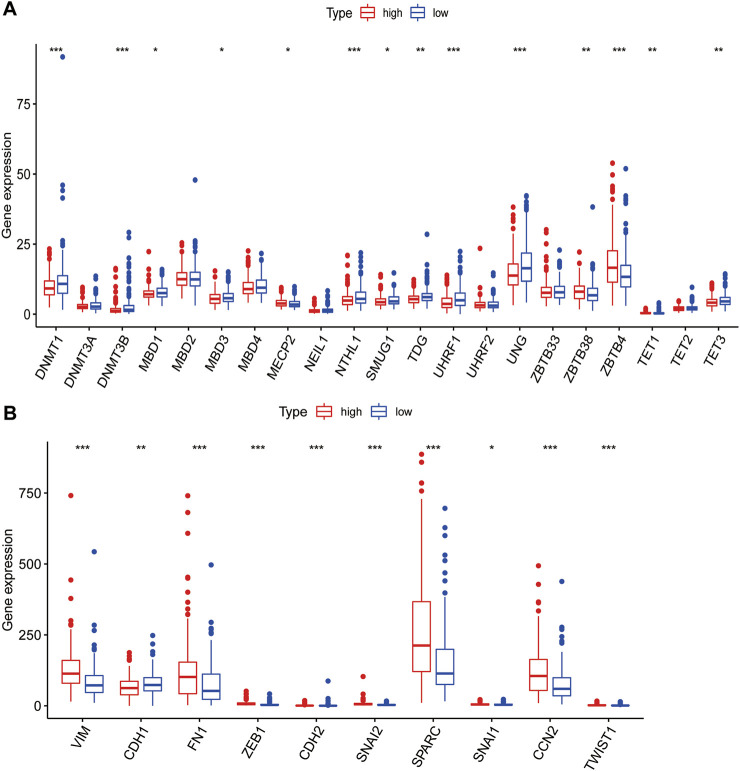
Correlation analysis of PLT-related lncRNAs with methylation and EMT. **(A)** Differences of 5mC Regulator expression between patients in the high- and low-risk groups. **(B)** Differences of EMT-related gene expression levels among high- and low-risk groups.

DNA methylation and lncRNA regulation are generally considered to be important factors in cell differentiation and development (Tang, 2018). Some studies indicated that DNA methylation at the same locus is associated with PLT activation variability in well-defined populations (Izzi et al., 2019). DNA methylation involved in general research mainly refers to the methylation process that occurs at the 5th-carbon atom of cytosine in CpG dinucleotides, a product also called 5-methylcytosine (5mC), which is the earliest methylation type excavated in eukaryotes (Ye and Li, 2014). As one of the important epigenetic markers, 5mC has a significant effect on various physiological and pathological processes (Ye and Li, 2014). Next, we explored whether there is a link between the risk model and DNA methylation. We analyzed the relationship between these 5mC regulators (DNMT1, DNMT3A, DNMT3B, MBD1, MBD2, MBD3, MBD4, MECP2, NEIL1, NTHL1, SMUG1, TDG, UHRF1, UHRF2, UNG, ZBTB33, ZBTB38, Z BTB4, TET1, TET2, TET3) in high- and low-risk groups (Chen et al., 2020). We discovered that most of the regulators were different between high- and low-risk groups (Figure 7A, *p* < 0.05), indicating that the risk model we constructed is correlated with DNA methylation. This result suggests that DNA methylation is one of the major biological characteristics of the high-risk group.

PLT could produce TGFβ, a cytokine highly related to EMT, which has an extremely important role in EMT(13). Therefore, we tried to explore the correlation between the risk model and EMT markers. EMT-related genes came from EMTome. Ten genes (VIM, CDH1, FN1, ZEB1, CDH2, SNAI2, SPARC, SNAI1, CCN2 and TWIST1) were selected for correlation analysis. We found that all the EMT-related genes we picked were significantly correlated with the risk model and the high-risk group patients had higher EMT gene expression (Figure 7B). The results indicated a strong correlation between the risk model and EMT, which may explain the poor prognosis of high-risk groups.

### Construction and assessment of the novel nomogram

**FIGURE 8:**
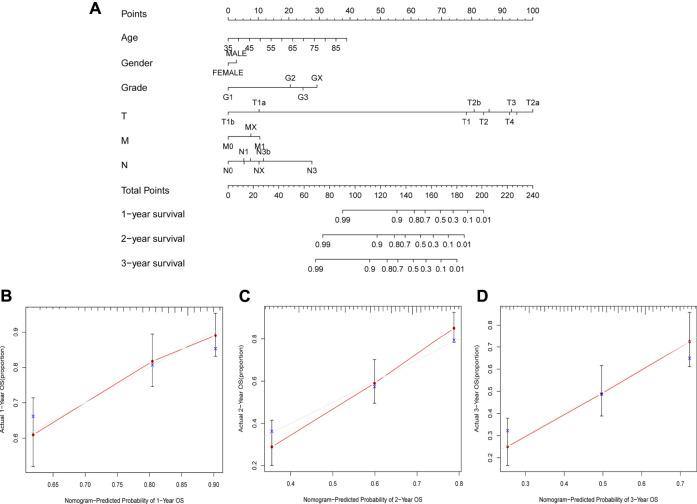
Construction and Assessment of the Novel Nomogram. **(A)** The nomogram that predicted 1 -, 2 -, and 3-year survival probabilities. **(B**–**D)** The calibration curve for 1 -, 2 -, and 3-year OS.

We also used 1-year, 2-year, and 3-year calibration charts to prove that the nomogram was in good agreement with the prediction of 1-, 2-, and 3-year OS (Figure 8A). Nomogram including risk grade and clinical risk characteristics were used to predict the incidence of OS at 1-, 2-, and 3-year. The risk level of the prognostic model showed outstanding predictive power in the nomogram compared to clinical factors (Figure 8A). The observed ratios of 1-year, 2-year, and 3-year OS showed definitive agreement with the predicted ratios (Figures 8B–D).

## Discussion

More and more studies have been conducted on lncRNAs in recent years, which have an important role in cancer progression (Chandra Gupta and Nandan Tripathi, 2017). However, there are few studies on the role of lncRNAs in GC. The study of PLT-related lncRNAs can provide a new direction for exploring GC pathogenesis and targeted therapy. It is particularly important to study the prognostic significance of PLT-related lncRNAs in GC. Over the years, research on the effect of lncRNAs and PLT in tumors has gradually become a hot topic in medical research.

Currently, growing evidence suggests that PLT play an important part in the occurrence and development of GC. Activated PLT can promote thrombus formation, thereby accelerating tumor progression (Suzuki-Inoue, 2019). Therefore, exploring the mechanism of PLT activation in the progression of GC has an important meaning in improving the survival rate of GC patients and improving the effect of immunotherapy. Molecular markers associated with PLT activation may also have an important role in predicting the clinical risk and prognosis of GC patients. Xie *et al.* proposed a novel PLT-related gene signature as a practical tool for patients with triple-negative breast cancer (TNBC) with independent value in assessing clinical prognosis (Xie et al., 2021). In addition, there are more and more research on the effect of lncRNAs in tumor progression, and they have attracted more and more attention (Li et al., 2016; Wang et al., 2022). A previous study has shown that TEPs derived lncRNAs occupy an important position in the diagnosis and treatment of colorectal cancer and may elucidate the underlying molecular mechanism of PLT-tumor cell interaction, which may be related to circulating lncRNAs in the blood (Ye et al., 2021). Therefore, both PLT and lncRNAs are closely related to the occurrence and development of GC. Still, there is little research on the role of PLT-related lncRNAs in the prognosis of GC patients. Xu *et al.* established an m6A-related lncRNAs model and confirmed the model’s important effect in predicting the prognosis of patients with lung adenocarcinoma (LUAD), providing guidance for the immunotherapy of patients with LUAD (Xu et al., 2021). Furthermore, a recent study on the effect of autophagy on GC constructed a prognostic model containing five autophagy-related lncRNAs, indicating the key role of autophagy-related lncRNAs in GC and suggesting that these lncRNAs may be effective targets for immunotherapy point (Chen et al., 2021a). In addition, models of ferroptosis-related lncRNAs, necroptosis-related lncRNAs, and pyroptosis-related lncRNAs have also been established, providing new targets for the study of the molecular mechanism, and the immunotherapy of malignant tumors (Song et al., 2021; Xiao et al., 2021; Zhao et al., 2021). In this study, we firstly constructed an independent prognostic model based on PLT-related lncRNAs.

We first extracted and identified 848 PLT -related lncRNAs from the TCGA database and performed a series of analytical validations to explore the value of PLT-related lncRNAs in GC prognosis. We verified the prognostic value of 18 PLT-related lncRNAs in GC by univariate COX regression analysis. By univariate COX regression analysis, we verified the prognostic value of 18 PLT-related lncRNAs in GC. Seven PLT-related lncRNAs (AL355574.1, LINC01697, AC002401.4, AC129507.1, AL513123.1, LINC01094, AL356417.2) were identified by LASSO regression analysis and used to construct the prognostic model for predicting OS in GC patients. AL355574.1 was identified as a protective lncRNA associated with autophagy in GC, which can be used as a promising therapeutic target for immunotherapy in GC patients (Chen et al., 2021a). Zhang *et al.* pointed out that LINC01697, as a ceRNA, could be used as a biomarker for the prognosis of GC patients and was up-regulated in GC cells, while its knockdown can inhibit the proliferation of GC cells (Zhang et al., 2021). Moreover, Li *et al.* suggested that LINC01697 as a prognostic biomarker for oral squamous cell carcinoma (Li et al., 2020a; Zha et al., 2021). Zha and others showed AC129507.1 as a DElncRNA was upregulated in GC and significantly associated with the prognosis of GC patients (Zha et al., 2021). Sun *et al.* found that AL513123.1 was upregulated in a high-risk group and could be used as a DElncRNA closely related to the prognosis of breast cancer (BRCA) (Sun et al., 2019). In addition, Tuersong *et al.* obtained similar results, pointing out that AL513123.1 in BRCA may be involved in the regulation of the complex ceRNA network and identified as a potential prognostic biomarker and therapeutic target for BRCA diagnosis and treatment (Tuersong et al., 2019). LINC01094 is associated with the prognosis of ovarian cancer, pancreatic cancer, glioma, and renal clear cell carcinoma (Xu et al., 2020a; Jiang et al., 2020; Chen et al., 2021b; Luo et al., 2021; Liu et al., 2022). Li *et al.* found that AL356417.2, as an immune-related lncRNA in BRCA, is closely associated with the prognosis of BRCA and can be used as a prognostic molecular marker and immunotherapy target for BRCA patients (Li et al., 2020b). In this study we discovered and verified AC002401 for the first time. Therefore, the seven PLT-related lncRNAs obtained in our study may also become the important biomarkers and therapeutic targets for GC and even other cancer types.

We divided patients into high- and low-risk groups according to the risk scores of the model and evaluated the mechanism of regulating GC progression by GSEA. The GSEA results indicated that the complement and coagulation pathway was the most upregulated gene-enriched signaling pathway in a high-risk group. In addition, immune-related pathways were also enriched, such as ECM receptor interactions. The complement system participates in multiple pathological processes such as thrombotic diseases, immune responses, autoimmune diseases, and cancer (Afshar-Kharghan, 2017). Firstly, it is involved in various tumorigenesis and cancer progression stages by mediating inflammatory responses. Secondly, complement activation may have a role by modulating T cell response to a tumor. Markiewski *et al.* showed that activation of the classical complement pathway promotes *in situ* tumor growth in mice (Markiewski et al., 2008). The immunomodulatory effect of the classical complement pathway activated in the tumor can promote tumor growth. In addition, we also noted that the ECM receptor interaction pathway was significantly enriched in a high-risk group. The role of ECM has been demonstrated in several cancers. Bao *et al.* showed that ECM-related proteins or genes might be potential biomarkers for breast cancer diagnosis and treatment (Bao et al., 2019). Studies have also shown that ECM participates in the invasion and metastasis of GC and promotes EMT in colorectal cancer (Rahbari et al., 2016; Yan et al., 2018).

Because the signal pathways enriched in this model are concentrated on immune-related signal pathways, the correlation between high- and low-risk groups and TME was analyzed. It was found that the model was closely related to immune cell infiltration. CIBERSPOT algorithm was then used to calculate the correlation of different immune cell infiltration. We noted that M2 macrophages, monocytes, and dendritic cells resting had a significantly higher expression in a high-risk group, which indicates that high-risk patients have higher immune cell infiltration. As for the low-risk group, we observed more infiltration of B cell naive and T cell follicular helper. In addition, we found higher immune scores, stromal scores, and estimated scores in the high-risk group. This is consistent with the results of previous studies that high immune score, stromal score, and macrophage infiltration are associated with poor prognoses (Deng et al., 2020). NK cell consumption significantly promotes cancer metastasis in mice (Shimaoka et al., 2017). PLT have been found to protect tumor cells from NK cells, and this effect is mainly due to the transfer of PLT-derived MHC CLASS I molecules to tumor cells after the interaction between PLT and tumor cells, which reduces the anti-tumor reactivity of NK cells and thus avoids immune surveillance (Placke et al., 2012). MSI analysis showed that MSI-H patients would have a better immunotherapy prognosis. In conclusion, the immunotherapy response-related prediction marker showed that patients in the high-risk group had a better response to immunotherapy. Based on this analysis, we concluded that the risk model could contribute to identifying reliable molecular biomarkers for the immunotherapy of GC.

DNA methylation modification is the most common covalent modification method. Many recent studies have confirmed the correlation between methylation modification and malignancy (Xu et al., 2020b). 5mC is the only form of DNA methylation found in mammals, and 5mC methylation regulators are associated with tumor proliferation and metastasis (Huang et al., 2021b). Benedetta *et al.* reported that PLT-endothelial aggregation receptor 1 (PEAR1), driven by DNA methylation, is a marker of PLT activation variability (Izzi et al., 2019). However, our study confirmed a correlation between the risk model and DNA methylation. Our study found that most DNA methylation regulators were differentially expressed in high-risk and low-risk groups. These results showed that there might be an association between our findings and DNA methylation, which reflects an important biological feature of the model. EMT has been shown to play an important role in tumorigenesis, invasion, and metastasis (Nieto et al., 2016). This study found that the EMT markers we selected were differentially expressed in both high- and low-risk groups. The results indicate a correlation between the risk model and EMT, reflecting another important biological feature of the model. These results suggest that DNA methylation and EMT are responsible for the poor prognosis of high-risk patients.

However, further experiments are needed to prove the effect of PLT-related lncRNAs on the prognosis of GC and related molecular mechanisms. The related signaling pathways screened out in this study and the effectiveness of immunotherapy drugs should be further investigated. In this study, we only analyzed and validated the data in the TCGA database. Although we have carried out some experimental verification using the collected specimens, there may still be deviations and deficiencies. Therefore, the risk model we constructed needs more external data for verification. We plan to collect more clinical samples to further validate the value of these lncRNAs in a future study.

## Conclusion

In this study, we constructed a model containing seven PLT-related lncRNAs. This study provides new clues for predicting the prognosis of GC patients and may help to elucidate the process and mechanism of PLT-related lncRNAs. In addition, small-molecule drugs were found to target PLT-related lncRNAs, and risk models showed sensitivity in distinguishing GC patients who benefited from immunotherapy. Our study further explored the role of PLT-related lncRNAs in TME, drug screening, and immunotherapy prediction in GC, providing new directions and therapeutic targets for further research and clinical practice.

## Data Availability

The original contributions presented in the study are included in the article/Supplementary Material, further inquiries can be directed to the corresponding authors.

## References

[B1] Afshar-Kharghan V. (2017). The role of the complement system in cancer. J. Clin. Invest. 127 (3), 780–789. 10.1172/JCI90962 28248200 PMC5330758

[B2] Bao Y. Wang L. Shi L. Yun F. Liu X. Chen Y. (2019). Transcriptome profiling revealed multiple genes and ECM-receptor interaction pathways that may be associated with breast cancer. Cell. Mol. Biol. Lett. 24, 38. 10.1186/s11658-019-0162-0 31182966 PMC6554968

[B3] Cao M. Li H. Sun D. Chen W. (2020). Cancer burden of major cancers in China: A need for sustainable actions. Cancer Commun. 40 (5), 205–210. 10.1002/cac2.12025 PMC7667573 32359212

[B4] Chandra Gupta S. Nandan Tripathi Y. (2017). Potential of long non-coding RNAs in cancer patients: From biomarkers to therapeutic targets. Int. J. Cancer 140 (9), 1955–1967. 10.1002/ijc.30546 27925173

[B5] Chen B. Khodadoust M. S. Liu C. L. Newman A. M. Alizadeh A. A. (2018). Profiling tumor infiltrating immune cells with CIBERSORT. Methods Mol. Biol. 1711, 243–259. 10.1007/978-1-4939-7493-1_12 29344893 PMC5895181

[B6] Chen D. Wang M. Xu Y. Jiang X. Xiong L. Zhang L. (2021). A novel autophagy-related lncRNA prognostic signature associated with immune microenvironment and survival outcomes of gastric cancer patients. Int. J. Gen. Med. 14, 6935–6950. 10.2147/IJGM.S331959 34703297 PMC8541751

[B7] Chen H. Liu Y. Liu P. Dai Q. Wang P. (2021). LINC01094 promotes the invasion of ovarian cancer cells and regulates the Wnt/β-catenin signaling pathway by targeting miR-532-3p. Exp. Ther. Med. 22 (5), 1228. 10.3892/etm.2021.10662 34539824 PMC8438678

[B8] Chen Y. T. Shen J. Y. Chen D. P. Wu C. F. Guo R. Zhang P. P. (2020). Identification of cross-talk between m(6)A and 5mC regulators associated with onco-immunogenic features and prognosis across 33 cancer types. J. Hematol. Oncol. 13 (1), 22. 10.1186/s13045-020-00854-w 32188475 PMC7081591

[B9] Chong D. L. W. Trinder S. Labelle M. Rodriguez-Justo M. Hughes S. Holmes A. M. (2020). Platelet-derived transforming growth factor-β1 promotes keratinocyte proliferation in cutaneous wound healing. J. Tissue Eng. Regen. Med. 14 (4), 645–649. 10.1002/term.3022 32068954 PMC7216944

[B10] Deng X. Lin D. Zhang X. Shen X. Yang Z. Yang L. (2020). Profiles of immune-related genes and immune cell infiltration in the tumor microenvironment of diffuse lower-grade gliomas. J. Cell. Physiol. 235 (10), 7321–7331. 10.1002/jcp.29633 32162312

[B11] Garmi N. Nasrallah S. Baram Y. Katz A. Koren A. First M. (2020). Platelets and breast cancer. Isr. Med. Assoc. J. 22 (10), 613–617. 33070484

[B12] Gns H. S. Gr S. Murahari M. Krishnamurthy M. (2019). An update on drug repurposing: Re-Written saga of the drug's fate. Biomed. Pharmacother. = Biomedecine Pharmacother. 110, 700–716. 10.1016/j.biopha.2018.11.127 30553197

[B13] Guo Y. Cui W. Pei Y. Xu D. (2019). Platelets promote invasion and induce epithelial to mesenchymal transition in ovarian cancer cells by TGF-β signaling pathway. Gynecol. Oncol. 153 (3), 639–650. 10.1016/j.ygyno.2019.02.026 30928020

[B14] Heldin C. H. Vanlandewijck M. Moustakas A. (2012). Regulation of EMT by TGFβ in cancer. FEBS Lett. 586 (14), 1959–1970. 10.1016/j.febslet.2012.02.037 22710176

[B15] Hu K. (2020). Become competent within one day in generating boxplots and violin plots for a novice without prior R experience. Methods Protoc. 3 (4), E64. 10.3390/mps3040064 32977580 PMC7712237

[B16] Huang Y. Yang Z. Huang C. Jiang X. Yan Y. Zhuang K. (2021). Identification of N6-methylandenosine-related lncRNAs for subtype identification and risk stratification in gastric adenocarcinoma. Front. Oncol. 11, 725181. 10.3389/fonc.2021.725181 34646770 PMC8504261

[B17] Huang Z. Pan J. Wang H. Du X. Xu Y. Wang Z. (2021). Prognostic significance and tumor immune microenvironment heterogenicity of m5C RNA methylation regulators in triple-negative breast cancer. Front. Cell Dev. Biol. 9, 657547. 10.3389/fcell.2021.657547 33928086 PMC8076743

[B18] Izzi B. Gianfagna F. Yang W. Y. Cludts K. De Curtis A. Verhamme P. (2019). Variation of PEAR1 DNA methylation influences platelet and leukocyte function. Clin. Epigenetics 11 (1), 151. 10.1186/s13148-019-0744-8 31665082 PMC6820903

[B19] Jiang P. Gu S. Pan D. Fu J. Sahu A. Hu X. (2018). Signatures of T cell dysfunction and exclusion predict cancer immunotherapy response. Nat. Med. 24 (10), 1550–1558. 10.1038/s41591-018-0136-1 30127393 PMC6487502

[B20] Jiang Y. Zhang H. Li W. Yan Y. Yao X. Gu W. (2020). FOXM1-Activated LINC01094 promotes clear cell renal cell carcinoma development via MicroRNA 224-5p/CHSY1. Mol. Cell. Biol. 40 (3), e00357-19. 10.1128/MCB.00357-19 31767633 PMC6965037

[B21] Kim J. Hwang I. C. (2020). Drawing guidelines for receiver operating characteristic curve in preparation of manuscripts. J. Korean Med. Sci. 35 (24), e171. 10.3346/jkms.2020.35.e171 32567255 PMC7308134

[B22] Kim S. Chen J. Cheng T. Gindulyte A. He J. He S. (2021). PubChem in 2021: New data content and improved web interfaces. Nucleic Acids Res. 49 (D1), D1388–D1395. 10.1093/nar/gkaa971 33151290 PMC7778930

[B23] Labelle M. Begum S. Hynes R. O. (2011). Direct signaling between platelets and cancer cells induces an epithelial-mesenchymal-like transition and promotes metastasis. Cancer Cell 20 (5), 576–590. 10.1016/j.ccr.2011.09.009 22094253 PMC3487108

[B24] Li J. Meng H. Bai Y. Wang K. (2016). Regulation of lncRNA and its role in cancer metastasis. Oncol. Res. 23 (5), 205–217. 10.3727/096504016X14549667334007 27098144 PMC7838649

[B25] Li Y. Cao X. Li H. (2020). Identification and validation of novel long non-coding RNA biomarkers for early diagnosis of oral squamous cell carcinoma. Front. Bioeng. Biotechnol. 8, 256. 10.3389/fbioe.2020.00256 32351944 PMC7174591

[B26] Li Z. Li Y. Wang X. Yang Q. (2020). Identification of a six-immune-related long non-coding RNA signature for predicting survival and immune infiltrating status in breast cancer. Front. Genet. 11, 680. 10.3389/fgene.2020.00680 32733537 PMC7358358

[B27] Lin A. Zhang J. Luo P. (2020). Crosstalk between the MSI status and tumor microenvironment in colorectal cancer. Front. Immunol. 11, 2039. 10.3389/fimmu.2020.02039 32903444 PMC7435056

[B28] Liu L. Xu Q. Xiong Y. Deng H. Zhou J. (2022). LncRNA LINC01094 contributes to glioma progression by modulating miR-224-5p/CHSY1 axis. Hum. Cell 35 (1), 214–225. 10.1007/s13577-021-00637-6 34716872

[B29] Livak K. J. Schmittgen T. D. (2001). Analysis of relative gene expression data using real-time quantitative PCR and the 2(-Delta Delta C(T)) Method. Methods (San Diego, Calif. 25 (4), 402–408. 10.1006/meth.2001.1262 11846609

[B30] Luo C. Lin K. Hu C. Zhu X. Zhu J. Zhu Z. (2021). LINC01094 promotes pancreatic cancer progression by sponging miR-577 to regulate LIN28B expression and the PI3K/AKT pathway. Mol. Ther. Nucleic Acids 26, 523–535. 10.1016/j.omtn.2021.08.024 34631282 PMC8479296

[B31] Markiewski M. M. DeAngelis R. A. Benencia F. Ricklin-Lichtsteiner S. K. Koutoulaki A. Gerard C. (2008). Modulation of the antitumor immune response by complement. Nat. Immunol. 9 (11), 1225–1235. 10.1038/ni.1655 18820683 PMC2678913

[B32] Meikle C. K. Meisler A. J. Bird C. M. Jeffries J. A. Azeem N. Garg P. (2020). Platelet-T cell aggregates in lung cancer patients: Implications for thrombosis. PloS one 15 (8), e0236966. 10.1371/journal.pone.0236966 32776968 PMC7416940

[B33] Nieto M. A. Huang R. Y. Jackson R. A. Thiery J. P. (2016). Emt: 2016. Cell 166 (1), 21–45. 10.1016/j.cell.2016.06.028 27368099

[B34] Oh S. E. Seo J. E. An J. Y. Lee J. H. Sohn T. S. Bae J. M. (2019). Prognostic impact of increased perioperative platelet count in gastric cancer patients. J. Surg. Res. 242, 296–303. 10.1016/j.jss.2019.04.052 31125843

[B35] Placke T. Örgel M. Schaller M. Jung G. Rammensee H. G. Kopp H. G. (2012). Platelet-derived MHC class I confers a pseudonormal phenotype to cancer cells that subverts the antitumor reactivity of natural killer immune cells. Cancer Res. 72 (2), 440–448. 10.1158/0008-5472.CAN-11-1872 22127925

[B36] Plantureux L. Mège D. Crescence L. Carminita E. Robert S. Cointe S. (2020). The interaction of platelets with colorectal cancer cells inhibits tumor growth but promotes metastasis. Cancer Res. 80 (2), 291–303. 10.1158/0008-5472.CAN-19-1181 31727628

[B37] Rahbari N. N. Kedrin D. Incio J. Liu H. Ho W. W. Nia H. T. (2016). Anti-VEGF therapy induces ECM remodeling and mechanical barriers to therapy in colorectal cancer liver metastases. Sci. Transl. Med. 8 (360), 360ra135. 360ra135. 10.1126/scitranslmed.aaf5219 PMC5457741 27733559

[B38] Ritchie M. E. Phipson B. Wu D. Hu Y. Law C. W. Shi W. (2015). Limma powers differential expression analyses for RNA-sequencing and microarray studies. Nucleic Acids Res. 43 (7), e47. 10.1093/nar/gkv007 25605792 PMC4402510

[B39] Rudzinski J. K. Govindasamy N. P. Lewis J. D. Jurasz P. (2020). The role of the androgen receptor in prostate cancer-induced platelet aggregation and platelet-induced invasion. J. Thromb. Haemost. 18 (11), 2976–2986. 10.1111/jth.15020 32692888

[B40] Sabrkhany S. Griffioen A. W. Oude Egbrink M. G. (2011). The role of blood platelets in tumor angiogenesis. Biochim. Biophys. Acta 1815 (2), 189–196. 10.1016/j.bbcan.2010.12.001 21167916

[B41] Schlesinger M. (2018). Role of platelets and platelet receptors in cancer metastasis. J. Hematol. Oncol. 11 (1), 125. 10.1186/s13045-018-0669-2 30305116 PMC6180572

[B42] Shimaoka H. Takeno S. Maki K. Sasaki T. Hasegawa S. Yamashita Y. (2017). A cytokine signal inhibitor for rheumatoid arthritis enhances cancer metastasis via depletion of NK cells in an experimental lung metastasis mouse model of colon cancer. Oncol. Lett. 14 (3), 3019–3027. 10.3892/ol.2017.6473 28928840 PMC5588136

[B43] Siegel R. L. Miller K. D. Fuchs H. E. Jemal A. (2021). Cancer statistics, 2021. Ca. Cancer J. Clin. 71 (1), 7–33. 10.3322/caac.21654 33433946

[B44] Simon N. Friedman J. Hastie T. Tibshirani R. (2011). Regularization paths for cox's proportional hazards model via coordinate descent. J. Stat. Softw. 39 (5), 1–13. 10.18637/jss.v039.i05 PMC4824408 27065756

[B45] Singla T. Singla G. Ranga S. Singla S. Arora R. (2020). Role of platelet aggregation in metastatic breast cancer patients. Indian J. Pathol. Microbiol. 63 (4), 564–569. 10.4103/IJPM.IJPM_817_19 33154306

[B46] Song W. Ren J. Xiang R. Kong C. Fu T. (2021). Identification of pyroptosis-related subtypes, the development of a prognosis model, and characterization of tumor microenvironment infiltration in colorectal cancer. Oncoimmunology 10 (1), 1987636. 10.1080/2162402X.2021.1987636 34676149 PMC8526024

[B47] Song Z. Wu Y. Yang J. Yang D. Fang X. (2017). Progress in the treatment of advanced gastric cancer. Tumour Biol. 39 (7), 1010428317714626. 10.1177/1010428317714626 28671042

[B48] Subramanian A. Narayan R. Corsello S. M. Peck D. D. Natoli T. E. Lu X. (2017). A next generation connectivity map: L1000 platform and the first 1, 000, 000 profiles. Cell 171 (6), 1437–1452. e17. 10.1016/j.cell.2017.10.049 29195078 PMC5990023

[B49] Subramanian A. Tamayo P. Mootha V. K. Mukherjee S. Ebert B. L. Gillette M. A. (2005). Gene set enrichment analysis: A knowledge-based approach for interpreting genome-wide expression profiles. Proc. Natl. Acad. Sci. U. S. A. 102 (43), 15545–15550. 10.1073/pnas.0506580102 16199517 PMC1239896

[B50] Sun M. Wu D. Zhou K. Li H. Gong X. Wei Q. (2019). An eight-lncRNA signature predicts survival of breast cancer patients: A comprehensive study based on weighted gene co-expression network analysis and competing endogenous RNA network. Breast Cancer Res. Treat. 175 (1), 59–75. 10.1007/s10549-019-05147-6 30715658

[B51] Suzuki-Inoue K. (2019). Platelets and cancer-associated thrombosis: Focusing on the platelet activation receptor CLEC-2 and podoplanin. Blood 134 (22), 1912–1918. 10.1182/blood.2019001388 31778548

[B52] Tan Z. (2019). Recent advances in the surgical treatment of advanced gastric cancer: A review. Med. Sci. Monit. 25, 3537–3541. 10.12659/MSM.916475 31080234 PMC6528544

[B53] Tang B. (2018). Inference of crosstalk effects between DNA methylation and lncRNA regulation in NSCLC. Biomed. Res. Int. 2018, 7602794. 10.1155/2018/7602794 30035125 PMC6035813

[B54] Tuersong T. Li L. Abulaiti Z. Feng S. (2019). Comprehensive analysis of the aberrantly expressed lncRNA-associated ceRNA network in breast cancer. Mol. Med. Rep. 19 (6), 4697–4710. 10.3892/mmr.2019.10165 31059025 PMC6522813

[B55] Vasaikar S. V. Deshmukh A. P. den Hollander P. Addanki S. Kuburich N. A. Kudaravalli S. (2021). EMTome: A resource for pan-cancer analysis of epithelial-mesenchymal transition genes and signatures. Br. J. Cancer 124 (1), 259–269. 10.1038/s41416-020-01178-9 33299129 PMC7782839

[B56] Wang H. Meng Q. Qian J. Li M. Gu C. Yang Y. (2022). Review: RNA-based diagnostic markers discovery and therapeutic targets development in cancer. Pharmacol. Ther. 234, 108123. 10.1016/j.pharmthera.2022.108123 35121000

[B57] Wang J. Zhang M. Zhou T. Zhao S. Su Z. Liu X. (2020). Role of platelet infiltration as independent prognostic marker for gastric adenocarcinomas. J. Clin. Lab. Anal. 34 (8), e23320. 10.1002/jcla.23320 32233046 PMC7439343

[B58] Whitehead M. J. McCanney G. A. Willison H. J. Barnett S. C. (2019). MyelinJ: An ImageJ macro for high throughput analysis of myelinating cultures. Bioinforma. Oxf. Engl. 35 (21), 4528–4530. 10.1093/bioinformatics/btz403 PMC6821319 31095292

[B59] Xiao S. Liu X. Yuan L. Wang F. (2021). A ferroptosis-related lncRNAs signature predicts prognosis and therapeutic response of gastric cancer. Front. Cell Dev. Biol. 9, 736682. 10.3389/fcell.2021.736682 34926441 PMC8674955

[B60] Xie J. Zou Y. Ye F. Zhao W. Xie X. Ou X. (2021). A novel platelet-related gene signature for predicting the prognosis of triple-negative breast cancer. Front. Cell Dev. Biol. 9, 795600. 10.3389/fcell.2021.795600 35096824 PMC8790231

[B61] Xu F. He L. Zhan X. Chen J. Xu H. Huang X. (2020). DNA methylation-based lung adenocarcinoma subtypes can predict prognosis, recurrence, and immunotherapeutic implications. Aging 12 (24), 25275–25293. 10.18632/aging.104129 33234739 PMC7803536

[B62] Xu F. Huang X. Li Y. Chen Y. Lin L. (2021). m(6 A-related lncRNAs are potential biomarkers for predicting prognoses and immune responses in patients with LUAD. Mol. Ther. Nucleic Acids 24, 780–791. 10.1016/j.omtn.2021.04.003 33996259 PMC8094594

[B63] Xu J. Zhang P. Sun H. Liu Y. (2020). LINC01094/miR-577 axis regulates the progression of ovarian cancer. J. Ovarian Res. 13 (1), 122. 10.1186/s13048-020-00721-9 33069244 PMC7568364

[B64] Yan P. He Y. Xie K. Kong S. Zhao W. (2018). *In silico* analyses for potential key genes associated with gastric cancer. PeerJ 6, e6092. 10.7717/peerj.6092 30568862 PMC6287586

[B65] Ye B. Li F. Chen M. Weng Y. Qi C. Xie Y. (2021). A panel of platelet-associated circulating long non-coding RNAs as potential biomarkers for colorectal cancer. Genomics 114 (1), 31–37. 10.1016/j.ygeno.2021.11.026 34843904

[B66] Ye B. Li F. Chen M. Weng Y. Qi C. Xie Y. (2022). A panel of platelet-associated circulating long non-coding RNAs as potential biomarkers for colorectal cancer. Genomics 114 (1), 31–37. 10.1016/j.ygeno.2021.11.026 34843904

[B67] Ye C. Li L. (2014). 5-hydroxymethylcytosine: A new insight into epigenetics in cancer. Cancer Biol. Ther. 15 (1), 10–15. 10.4161/cbt.27144 24253310 PMC3938512

[B68] Yoshihara K. Shahmoradgoli M. Martínez E. Vegesna R. Kim H. Torres-Garcia W. (2013). Inferring tumour purity and stromal and immune cell admixture from expression data. Nat. Commun. 4, 2612. 10.1038/ncomms3612 24113773 PMC3826632

[B69] Zaslavsky A. B. Adams M. P. Cao X. Maj T. Choi J. E. Stangl-Kremser J. (2020). Platelet PD-L1 suppresses anti-cancer immune cell activity in PD-L1 negative tumors. Sci. Rep. 10 (1), 19296. 10.1038/s41598-020-76351-4 33168847 PMC7652857

[B70] Zeltz C. Primac I. Erusappan P. Alam J. Noel A. Gullberg D. (2020). Cancer-associated fibroblasts in desmoplastic tumors: Emerging role of integrins. Semin. Cancer Biol. 62, 166–181. 10.1016/j.semcancer.2019.08.004 31415910

[B71] Zha Z. Zhang P. Li D. Liu G. Lu L. (2021). Identification and construction of a long noncoding RNA prognostic risk model for stomach adenocarcinoma patients. Dis. Markers 2021, 8895723. 10.1155/2021/8895723 33680217 PMC7929674

[B72] Zhang S. Li S. Guo J. L. Li N. Zhang C. N. Liu J. (2021). Integrated analysis of lncRNA-associated ceRNA network identifies two lncRNA signatures as a prognostic biomarker in gastric cancer. Dis. Markers 2021, 8886897. 10.1155/2021/8886897 34603561 PMC8479203

[B73] Zhao Z. Liu H. Zhou X. Fang D. Ou X. Ye J. (2021). Necroptosis-related lncRNAs: Predicting prognosis and the distinction between the cold and hot tumors in gastric cancer. J. Oncol. 2021, 6718443. 10.1155/2021/6718443 34790235 PMC8592775

