# Supplementary figures and images for: Screening and validation of platelet activation-related lncRNAs as potential biomarkers for prognosis and immunotherapy in gastric cancer patients
Source: Front Genet. 2022 Sep 14;13:965033. doi: 10.3389/fgene.2022.965033 (PMC9515443; doi:10.3389/fgene.2022.965033)

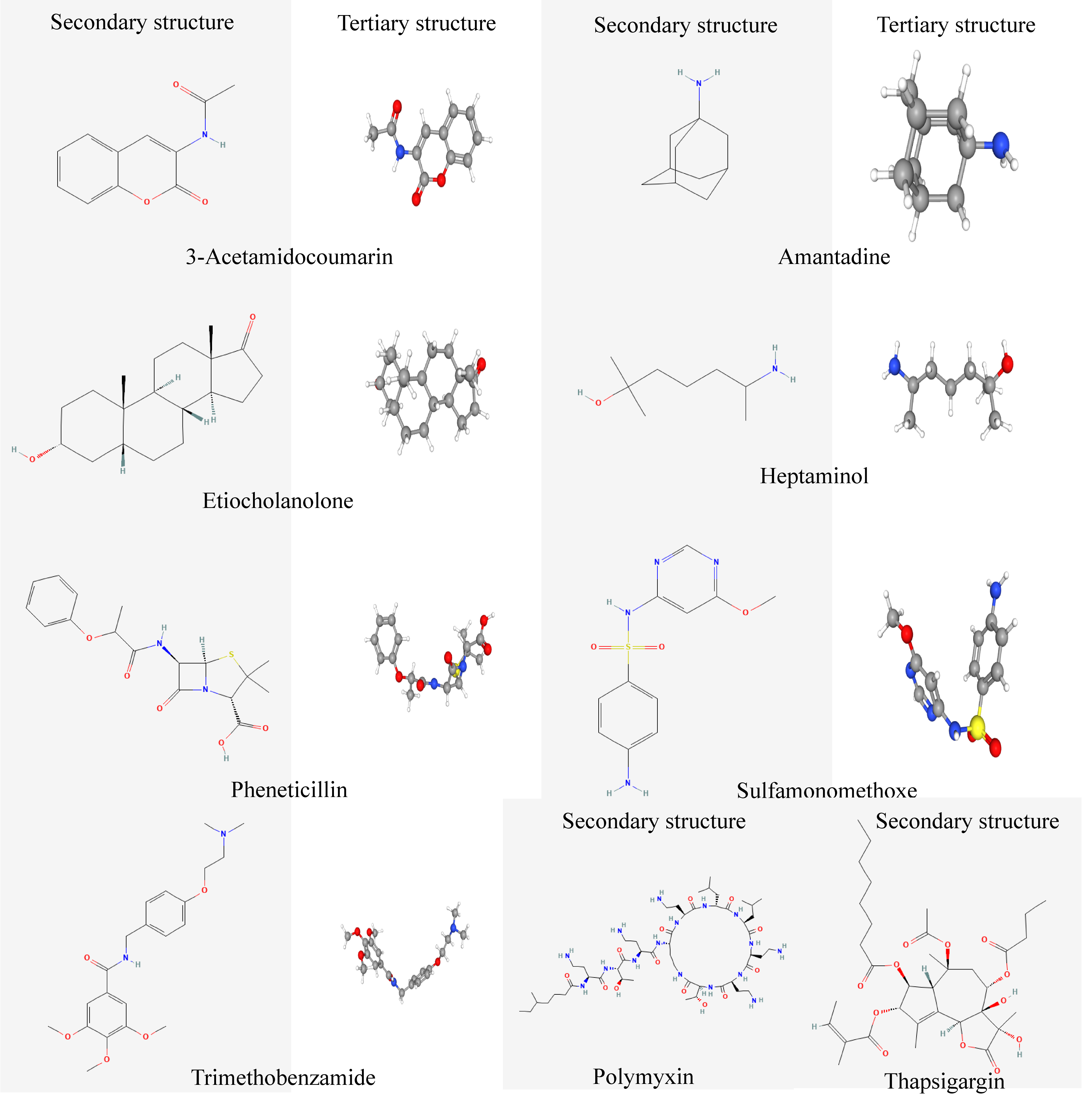

Supplement: Supplementary file 1 [file Image3.TIF]

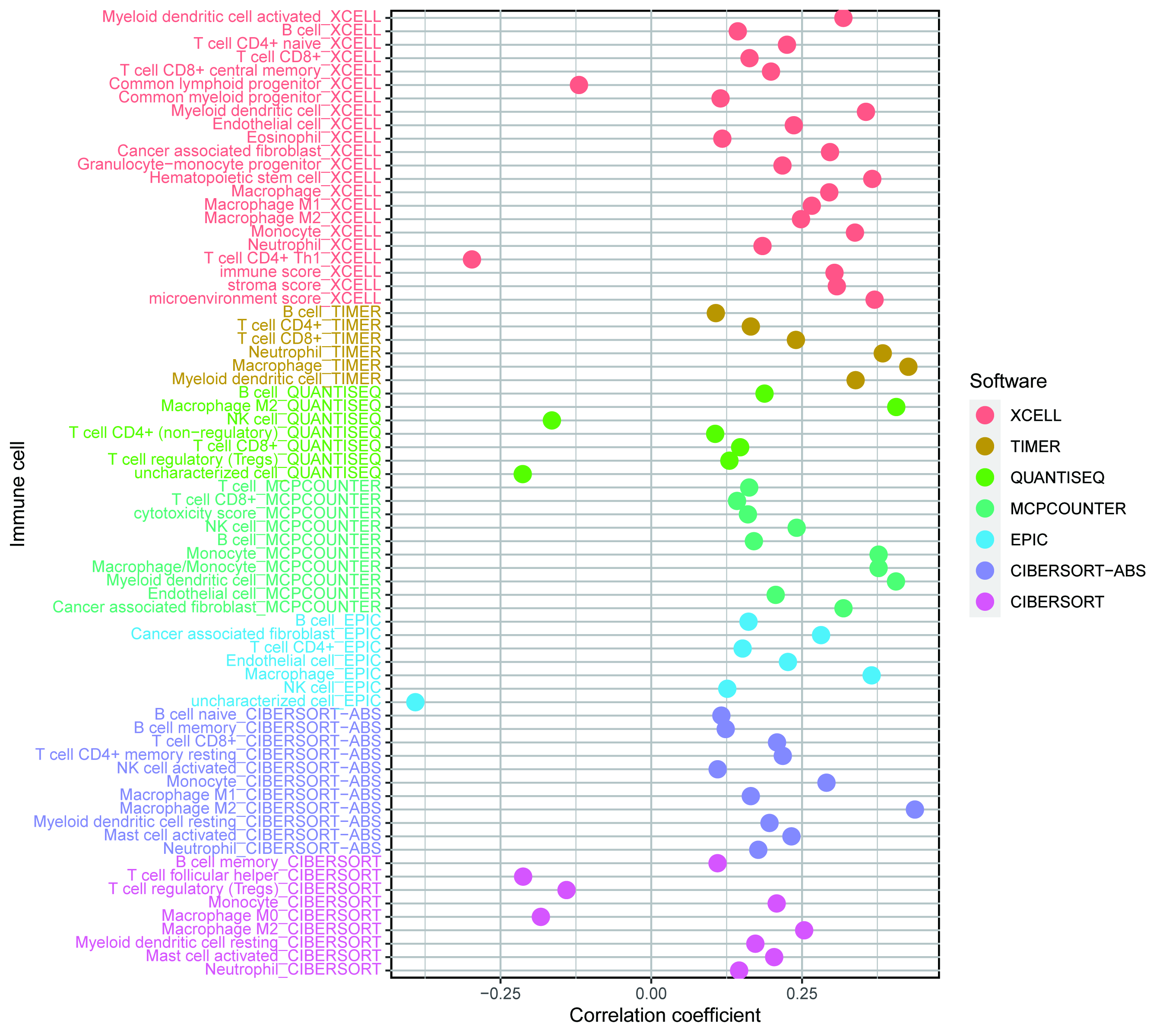

Supplement: Supplementary file 2 [file Image4.TIF]

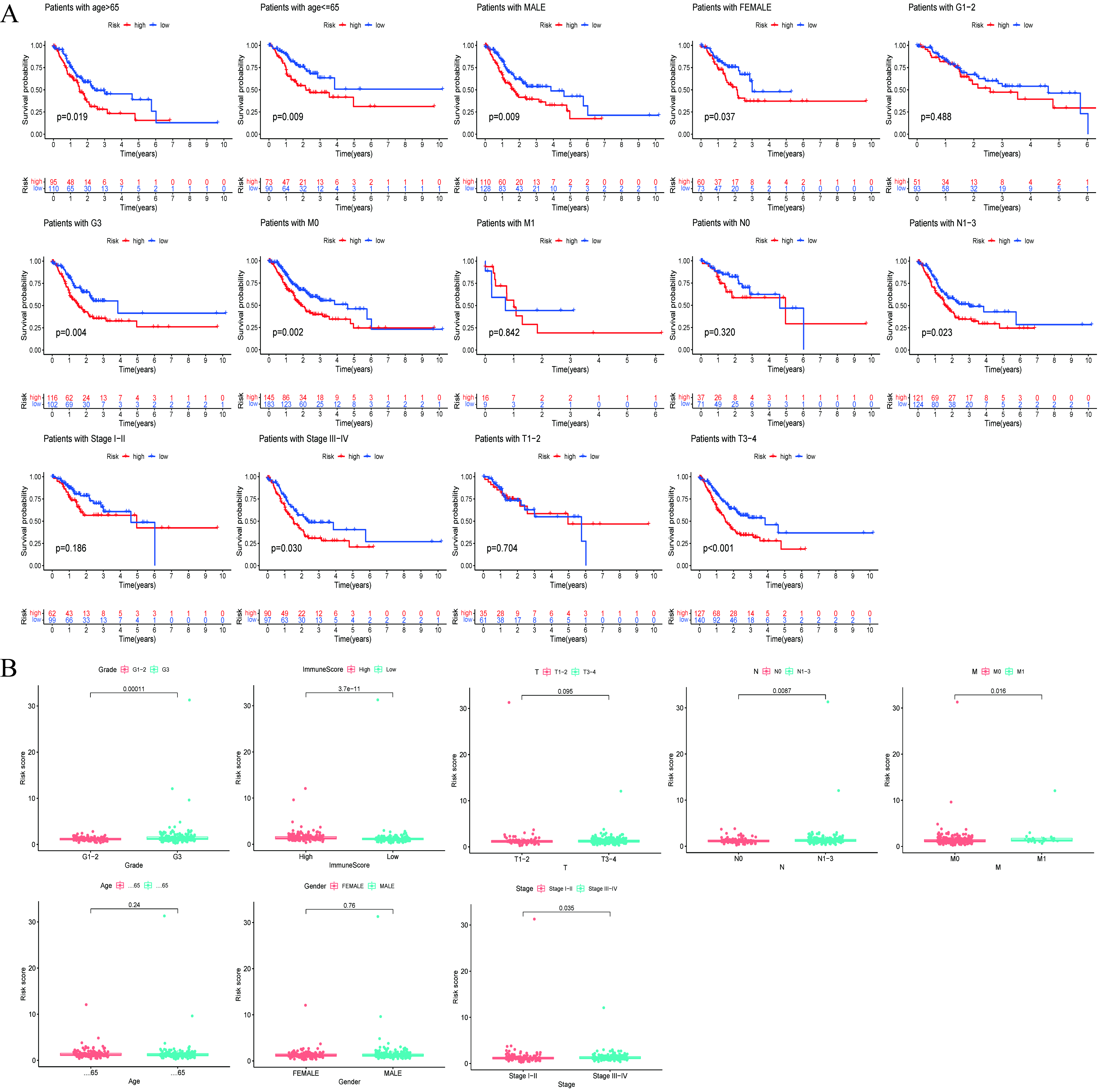

Supplement: Supplementary file 3 [file Image2.TIF]

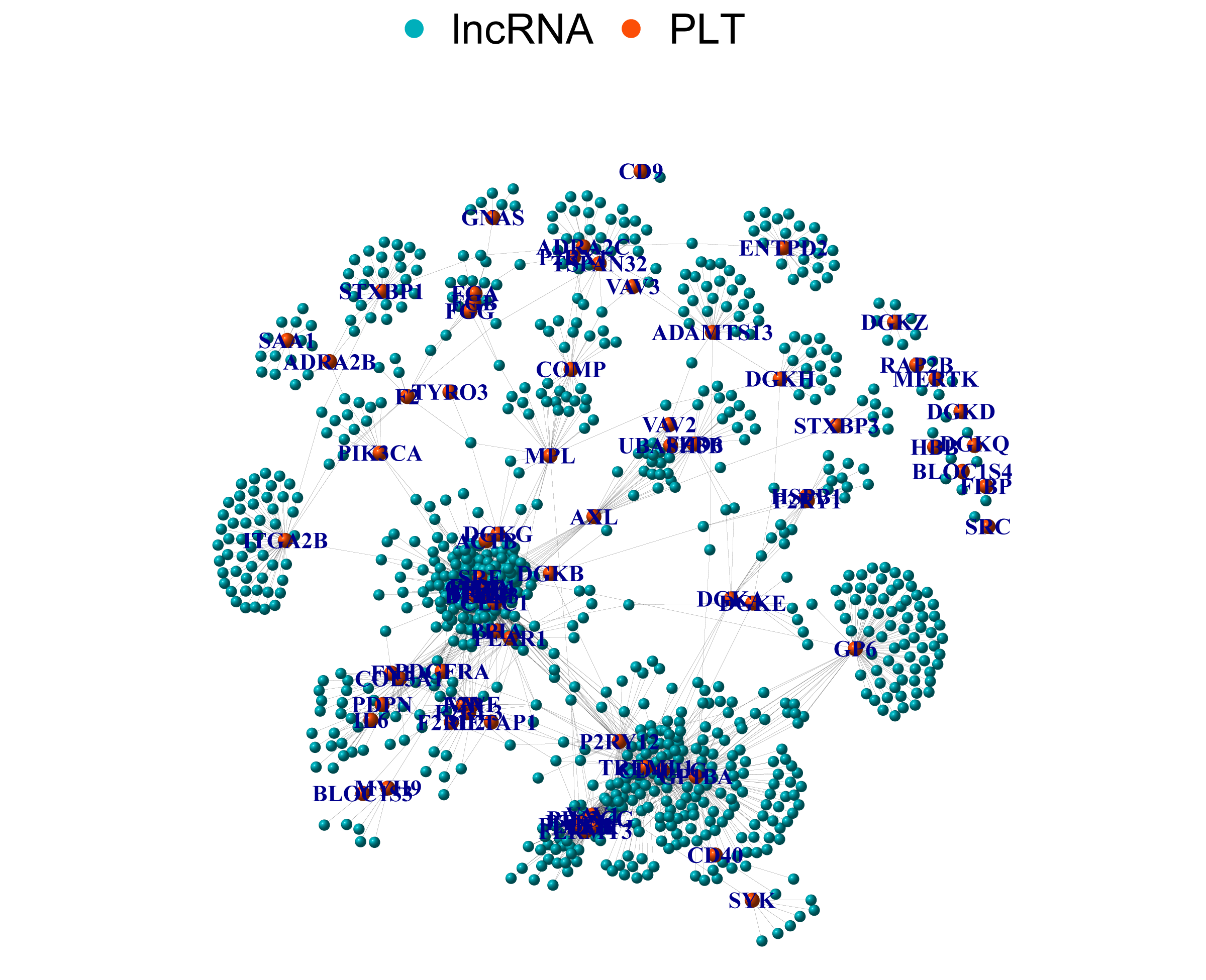

Supplement: Supplementary file 4 [file Image1.TIF]

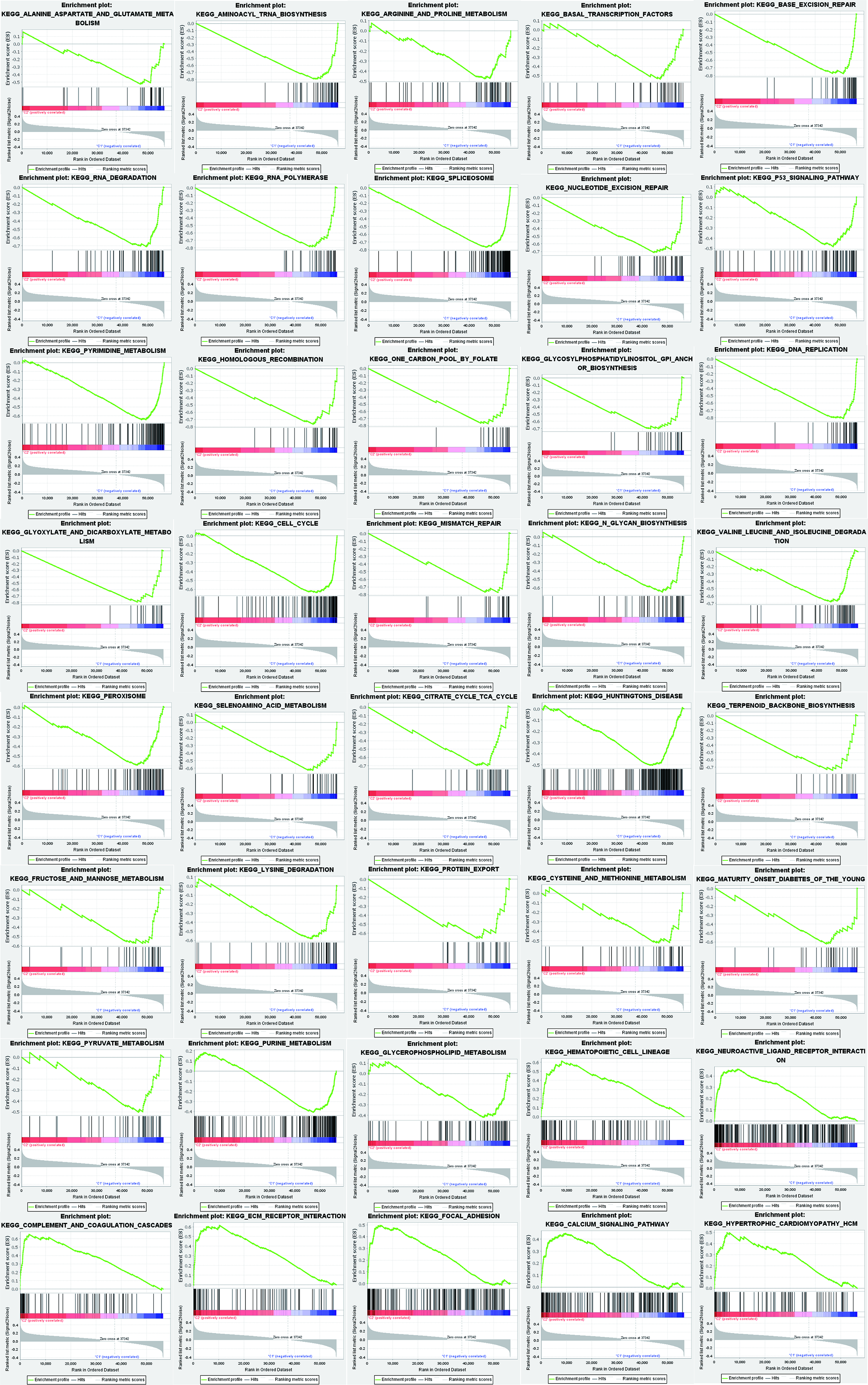

Supplement: Supplementary file 7 [file Image5.TIF]
