# Supplementary material for: Screening and validation of platelet activation-related lncRNAs as potential biomarkers for prognosis and immunotherapy in gastric cancer patients
Source: Front Genet. 2022 Sep 14;13:965033. doi: 10.3389/fgene.2022.965033 (PMC9515443; doi:10.3389/fgene.2022.965033)
Supplement: Supplementary file 5 [file Table2.DOCX]

**Supplementary Table S1.** Clinicopathological characteristics of the included patients

| Group | Gender | Age | Pathological Type | Grade | **T** | **N** | **M** |
| --- | --- | --- | --- | --- | --- | --- | --- |
| 1 | male | 67 | STAD | III | T3 | N1 | M0 |
| 2 | male | 66 | STAD | III | T2 | N1 | MO |
| 3 | male | 66 | STAD | III | T2 | N2 | MO |
| 4 | male | 58 | STAD | III | T3 | N2 | M0 |
| 5 | male | 62 | STAD | II | T4b | N0 | M0 |
| 6 | male | 78 | STAD | II | T4a | N0 | M0 |
| 7 | male | 89 | STAD | III | T4b | N1 | M0 |
| 8 | female | 48 | STAD | III | T4a | NO | M1 |
| 9 | male | 56 | STAD | II | T2 | N0 | M0 |
| 10 | female | 64 | STAD | III | T4a | N3 | M0 |

**Supplementary Table S2.** The primers used for qRT-PCR.

| Primer Name | Primer Sequence (5' -3') |
| --- | --- |
| AL355574.1-Forward Primer | GGAGGGCAGAGAGCAACGTA |
| AL355574.1-Reverse Primer | CGCCTCTACAGACAGCACTC |
| AC129507.1-Forward Primer | GGACAGATGGTGGGAGAGGAA |
| AC129507.1-Reverse Primer | ATGCTGACGAGGTTTGGAGAATC |
| AC002401.4-Forward Primer | CACCTCCTGGGATCTTGCTCTAA |
| AC002401.4-Reverse Primer | ACCGAACATAAAGCCTTGGACATC |
| AL356417.2-Forward Primer | CACCGAACACCAGTGCAGTAG |
| AL356417.2-Reverse Primer | GGAGAGAAGGAAGCCACGCAT |
| AL513123.1-Forward Primer | AAGACGGGTGATTTCTGCATTTC |
| AL513123.1-Reverse Primer | ACACAGGTAGCAACAACTCCAA |
| LINC01697-Forward Primer | GCCAATAAGATGTTCCTGCCTAAG |
| LINC01697-Reverse Primer | TGCTGTGCTGTGCTGTGAATA |
| LINC01094-Forward Primer | GTCAGGAGGAAGAAAGGAAGAGG |
| LINC01094-Reverse Primer | GGAGTGGACAAGTGGTCAAGT |
| β-Actin-Forward Primer | CTCGCTTCGGCAGCACA |
| β-Actin-Reverse Primer | AACGCTTCACGAATTTGCGT |

**Supplementary Table S4.** The results of GSEA in high-risk group.

|  | GS | NES | *P*-value | FDR q-val |
| --- | --- | --- | --- | --- |
| 1 | KEGG_COMPLEMENT_AND_COAGULATION_CASCADES | 1.97 | 0.004 | 0.134 |
| 2 | KEGG_HEMATOPOIETIC_CELL_LINEAGE | 1.91 | 0.01 | 0.124 |
| 3 | KEGG_NEUROACTIVE_LIGAND_RECEPTOR_INTERACTION | 1.86 | 0.002 | 0.131 |
| 4 | KEGG_ECM_RECEPTOR_INTERACTION | 1.8 | 0.017 | 0.17 |
| 5 | KEGG_CALCIUM_SIGNALING_PATHWAY | 1.69 | 0.021 | 0.297 |
| 6 | KEGG_HYPERTROPHIC_CARDIOMYOPATHY_HCM | 1.69 | 0.034 | 0.254 |
| 7 | KEGG_FOCAL_ADHESION | 1.67 | 0.029 | 0.25 |
| 8 | KEGG_VASCULAR_SMOOTH_MUSCLE_CONTRACTION | 1.66 | 0.035 | 0.234 |
| 9 | KEGG_CELL_ADHESION_MOLECULES_CAMS | 1.65 | 0.05 | 0.227 |
| 10 | KEGG_DILATED_CARDIOMYOPATHY | 1.63 | 0.043 | 0.225 |
| 11 | KEGG_ARRHYTHMOGENIC_RIGHT_VENTRICULAR_CARDIOMYOPATHY_ARVC | 1.51 | 0.085 | 0.415 |
| 12 | KEGG_CYTOKINE_CYTOKINE_RECEPTOR_INTERACTION | 1.51 | 0.073 | 0.387 |
| 13 | KEGG_ABC_TRANSPORTERS | 1.48 | 0.049 | 0.407 |

**Supplementary Table S5.** The results of GSEA in low-risk group.

|  | GS | NES | P-value | FDR q-val |
| --- | --- | --- | --- | --- |
| 1 | KEGG_SPLICEOSOME | -2.27 | 0.002 | 0.003 |
| 2 | KEGG_RNA_DEGRADATION | -2.19 | 0.002 | 0.004 |
| 3 | KEGG_RNA_POLYMERASE | -2.18 | 0 | 0.003 |
| 4 | KEGG_PYRIMIDINE_METABOLISM | -2.13 | 0.002 | 0.005 |
| 5 | KEGG_AMINOACYL_TRNA_BIOSYNTHESIS | -2.11 | 0 | 0.005 |
| 6 | KEGG_BASE_EXCISION_REPAIR | -2.11 | 0.002 | 0.005 |
| 7 | KEGG_NUCLEOTIDE_EXCISION_REPAIR | -2.07 | 0.004 | 0.008 |
| 8 | KEGG_HOMOLOGOUS_RECOMBINATION | -2.03 | 0.002 | 0.013 |
| 9 | KEGG_ONE_CARBON_POOL_BY_FOLATE | -1.97 | 0.002 | 0.021 |
| 10 | KEGG_GLYCOSYLPHOSPHATIDYLINOSITOL_GPI_ANCHOR_BIOSYNTHESIS | -1.96 | 0.008 | 0.02 |
| 11 | KEGG_GLYOXYLATE_AND_DICARBOXYLATE_METABOLISM | -1.96 | 0.004 | 0.019 |
| 12 | KEGG_DNA_REPLICATION | -1.94 | 0.006 | 0.021 |
| 13 | KEGG_CELL_CYCLE | -1.94 | 0.015 | 0.021 |
| 14 | KEGG_VALINE_LEUCINE_AND_ISOLEUCINE_DEGRADATION | -1.93 | 0.01 | 0.021 |
| 15 | KEGG_MISMATCH_REPAIR | -1.92 | 0.006 | 0.021 |
| 16 | KEGG_N_GLYCAN_BIOSYNTHESIS | -1.86 | 0.016 | 0.034 |
| 17 | KEGG_PEROXISOME | -1.85 | 0.004 | 0.036 |
| 18 | KEGG_SELENOAMINO_ACID_METABOLISM | -1.83 | 0.004 | 0.041 |
| 19 | KEGG_CITRATE_CYCLE_TCA_CYCLE | -1.78 | 0.035 | 0.055 |
| 20 | KEGG_TERPENOID_BACKBONE_BIOSYNTHESIS | -1.77 | 0.017 | 0.053 |
| 21 | KEGG_HUNTINGTONS_DISEASE | -1.75 | 0.023 | 0.059 |
| 23 | KEGG_LYSINE_DEGRADATION | -1.73 | 0.031 | 0.067 |
| 24 | KEGG_PROTEIN_EXPORT | -1.73 | 0.033 | 0.065 |
| 25 | KEGG_BASAL_TRANSCRIPTION_FACTORS | -1.71 | 0.033 | 0.07 |
| 26 | KEGG_FRUCTOSE_AND_MANNOSE_METABOLISM | -1.7 | 0.036 | 0.072 |
| 28 | KEGG_CYSTEINE_AND_METHIONINE_METABOLISM | -1.64 | 0.031 | 0.095 |
| 29 | KEGG_P53_SIGNALING_PATHWAY | -1.62 | 0.046 | 0.102 |
| 30 | KEGG_PYRUVATE_METABOLISM | -1.62 | 0.043 | 0.101 |
| 31 | KEGG_MATURITY_ONSET_DIABETES_OF_THE_YOUNG | -1.62 | 0.034 | 0.098 |
| 32 | KEGG_ALANINE_ASPARTATE_AND_GLUTAMATE_METABOLISM | -1.62 | 0.037 | 0.097 |
| 34 | KEGG_PURINE_METABOLISM | -1.59 | 0.036 | 0.11 |
| 35 | KEGG_ARGININE_AND_PROLINE_METABOLISM | -1.58 | 0.049 | 0.108 |
